# Targeting Aurora Kinases as Essential Cell‐Cycle Regulators to Deliver Multi‐Stage Antimalarials Against *Plasmodium Falciparum*

**DOI:** 10.1002/anie.202518493

**Published:** 2025-10-23

**Authors:** Henrico Langeveld, Keletso Maepa, Marché Maree, Jessica L. Thibaud, Nicolaas Salomane, Rosie Bridgwater, Mufuliat T. Famodimu, Luiz C. Godoy, Charisse Flerida A. Pasaje, Nonlawat Boonyalai, Mariana Laureano de Souza, Justin Fong, Tayla Rabie, Mariëtte van der Watt, Rensu P. Theart, Sonja Ghidelli‐Disse, Jacquin C. Niles, Marcus C. S. Lee, Elizabeth A. Winzeler, Michael J. Delves, Kelly Chibale, Kathryn J. Wicht, Lauren B. Coulson, Lyn‐Marié Birkholtz

**Affiliations:** ^1^ Department of Biochemistry, Genetics and Microbiology Hatfield Pretoria 0028 South Africa; ^2^ Institute for Sustainable Malaria Control University of Pretoria Hatfield Pretoria 0028 South Africa; ^3^ South African Medical Research Council Drug Discovery and Development Research Unit, Department of Chemistry and Institute of Infectious Disease and Molecular Medicine University of Cape Town Rondebosch, Cape Town 7701 South Africa; ^4^ Holistic Drug Discovery and Development (H3D) Centre University of Cape Town Rondebosch, Cape Town 7701 South Africa; ^5^ Department of Biochemistry Stellenbosch University Stellenbosch 7602 South Africa; ^6^ LSHTM Malaria Centre London School of Hygiene and Tropical Medicine London UK; ^7^ Department of Infection Biology Faculty of Infectious Tropical Diseases London School of Hygiene and Tropical Medicine London UK; ^8^ Department of Biological Engineering Massachusetts Institute of Technology Cambridge MA 02139 USA; ^9^ Division of Biological Chemistry and Drug Discovery Wellcome Centre for Anti‐Infectives Research University of Dundee Dundee UK; ^10^ Department of Pediatrics School of Medicine University of California San Diego CA 92093 USA; ^11^ Department of Electrical and Electronic Engineering Stellenbosch University Stellenbosch 7602 South Africa; ^12^ Cellzome GmbH, GSK Company Heidelberg Germany

**Keywords:** Anti‐cancer inhibitors, Antimalarial drug discovery, Aurora kinase, Cell cycle regulation, *Plasmodium*

## Abstract

Kinases play critical roles in the development and adaptation of *Plasmodium falciparum* and present novel opportunities for chemotherapeutic intervention. Mitotic kinases that regulate the proliferation of the parasites by controlling nuclear division, segregation, and cytokinesis. We evaluated the potential of human Aurora kinase (Aur) inhibitors to prevent *P. falciparum* development by targeting members of the Aurora‐related kinase (Ark) family in this parasite. Several human AurB inhibitors exhibited multistage potency (< 250 nM) against all proliferative stages of parasite development, including asexual blood stages, liver schizonts, and male gametes. The most potent compounds, hesperadin, TAE684, and AT83, exhibited > 1000x selectivity towards the parasite. Importantly, we identified *Pf*Ark1 as the principal vulnerable Ark family member, with specific inhibition of *Pf*Ark1 as the primary target for hesperadin. Hesperadin's whole‐cell and protein activity validates it as a unique *Pf*Ark1 tool compound. Inhibition of *Pf*Ark1 results in the parasite's inability to complete mitotic processes, presenting with unsegregated, multi‐lobed nuclei caused by aberrant microtubule organization. This suggests *Pf*Ark1 is the main Aur mitotic kinase in proliferative stages of *Plasmodium*, characterized by bifunctional AurA and B activity. This paves the way for drug‐discovery campaigns based on hesperadin targeting *Pf*Ark1.

## Graphical abstract

In this study, we repurposed human Aurora kinase‐specific inhibitors to identify potential antimalarial agents. Two inhibitors, hesperadin and TAE684, exhibited sub‐micromolar activity across multiple parasite stages, with hesperadin demonstrating significant potency and selectivity by specifically targeting *Pf*Ark1. Inhibition of *Pf*Ark1 resulted in anaphase‐like abnormalities, pointing to its role as the primary *Pf*Ark for cell division in malaria parasites.

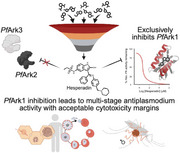

## Introduction

*Plasmodia spp*. parasites demonstrate pathogenic success due to their complex life cycle, alternating between non‐proliferative stages, where the cell cycle is quiescent, and stages characterized by rapid cell division events that lead to massive parasite population expansion^[^ ^1^, ^2^ ^]^ *Plasmodium falciparum*, the causative agent of the most severe form of malaria,^[^ ^3^ ^]^ undergoes three unique and specialized cell division events. During hepatic and intra‐erythrocytic schizogony within the human host, a haploid parasite undergoes multiple rounds of closed asynchronous mitosis and karyokinesis, followed by a singular and synchronized cytokinesis event to produce a segmented schizonts.^[^ ^4^, ^5^ ^]^ An equally unique cell division event occurs within the mosquito host, where male gametocytes (1n) undergo three rounds of rapid DNA replication (exflagellation) to generate eight flagellated male gametes (8n) in just ∼15 min.^[^ ^6^, ^7^ ^]^ The parasite's ability to undergo rapid asexual replication is a key factor in its pathogenic success but requires extraordinary control. A detailed mechanistic understanding of the role players in regulating the parasite's atypical cell cycle in *Plasmodium* could lead to novel antimalarial therapeutic agents.

Cell‐cycle machinery and regulators, such as protein kinases (PKs), are crucial for accurate progression through various checkpoints in mammalian cells. Several conserved Ser/Thr mitotic PKs are considered primary regulators of the mitotic process, including the “Never In Mitosis” kinases (NIMA/Neks), Polo‐like kinases, and Aurora kinases (Aur). The Aur family is highly conserved amongst eukaryotes, with members identified, amongst others, in yeasts (Ipl1), humans (*Hs*AurA, B, and C), *Toxoplasma gondii* (*Tg*Ark1–3),^[^ ^8^ ^]^ *Trypanosoma brucei* (*Tb*AUK1).^[^ ^9^ ^]^ In *P. falciparum*, three aurora‐related kinases exist (*Pf*Ark1–3).^[^ ^10^, ^11^ ^]^ Aur contributes to the assembly and disassembly of mitotic and meiotic centrosomes, regulating spindle‐pole structure and dynamics, chromosome segregation, and cellular fission during cytokinesis. Although Aur members are differentiated functionally depending on their localization, delocalization can cause moonlighting effects between *Hs*AurA and B,^[^ ^12^ ^]^ although direct compensation for the loss of activity is not evident.^[^ ^13^ ^]^ During mitosis, *Hs*AurA (“polar” Aur) localizes to the centrosome and spindle poles, and upon binding of microtubule‐associated protein TPX2, regulates centrosome maturation, separation, and microtubule spindle formation. *Hs*AurB as “equatorial” Aur (with INCENP, survivin, and borealin), forms the chromosome passenger complex (CPC) as master controller of cell division, localized to centromeres, kinetochores, and the spindle midzone, allowing AurB to govern chromosome condensation, kinetochore attachment, sister chromatid segregation, and cytokinesis.

*Pf*Arks are implicated in critically regulating cell‐cycle progression of *Plasmodium* based on 1) the essentiality of all three *Pf*Arks to asexual proliferation (schizogony),^[^ ^14^ ^]^ 2) the unique expression patterns of the *Pf*Arks during cell‐cycle arrest and re‐entry,^[^ ^15^ ^]^ and 3) distinct, highly specific, and exclusive spatiotemporal associations during ABS schizogony.^[^ ^16^ ^]^ Although *P. falciparum* lacks a canonical centrosome, it possesses a microtubule‐organizing center (MTOC) characterized by a centriolar plaque (CP) embedded in the nuclear envelope, with inner CP (intranuclear body) and outer CP domains (cytoplasmic body).^[^ ^17^ ^]^ The CPs harbor several validated centrosomal proteins, including centrin and γ‐tubulin, and facilitate microtubule (MT) nucleation. *Pf*Ark1 (PF3D7_0605300) and *Pf*Ark2 (PF3D7_0309200) are associated with MTOCs during schizogony,^[^ ^16^ ^]^ with *Pf*Ark1 localizing to the outer CP domains of duplicated MTOCs in nuclei primed for division (similar to a “polar” Aur^[^ ^10^ ^]^) while *Pf*Ark2 is additionally proposed to localize to kinetochores, akin to an “equatorial” Aur.^[^ ^16^ ^]^ *Pf*Ark3 (PF3D7_1356800) is found only in segmented nuclei associated with subpellicular microtubules (SPMTs) as cytosolic microtubules in merozoites, suggesting a role in cytokinesis.^[^ ^16^ ^]^

The deregulation of *Hs*Aurs (especially *Hs*AurA and *Hs*AurB) has been linked to cancer and tumorigenesis, making them attractive targets for anticancer therapeutic strategies.^[^ ^18^ ^]^ Several inhibitors selectively target either *Hs*AurA or *Hs*AurB or have dual or pan‐reactive abilities. *Hs*AurA inhibition leads to defects in mitotic spindle assembly and ultimately causes spindle checkpoint‐dependent mitotic arrest, cell cycle exit, and apoptosis.^[^ ^19^ ^]^ On the other hand, *Hs*AurB inhibition causes abnormal chromosome alignment and overrides the mitotic spindle checkpoint, causing polyploidy, failure of cytokinesis, and endoreduplication.^[^ ^20^ ^]^

Although several kinase families have been chemically and genetically validated as antimalarial targets (e.g., *Pf*PI4K,^[^ ^21^ ^]^ *Pf*PKG^[^ ^22^ ^],^ and *Pf*CLK3^[^ ^23^ ^]^), the unique requirement of Ark members for parasite‐proliferation processes has not been extensively explored to identify novel inhibitors specifically targeting this kinase family. Previous studies have shown that the *Hs*AurB‐specific inhibitor hesperadin exhibits potent in vitro activity against *P. falciparum, Trypanosoma brucei*, and *Leishmania donovani*.^[^ ^24^, ^25^, ^26^ ^]^ We also demonstrated that hesperadin treatment leads to mutations in *Pf*Ark1 that confer resistance.^[^ ^27^ ^]^ However, evidence of direct target engagement and inhibition of the *Pf*Ark members is lacking.

Here, we provide an in‐depth evaluation of *Pf*Ark inhibition and its influence on parasite survival. We systematically assessed *Hs*Aur inhibitors targeting all Aur classes to identify compounds that could be repurposed as antimalarials. We identify Ark inhibitors with multistage activity against proliferative stages of the parasite, including asexual blood stage (ABS) parasites, male gametes, and liver schizonts, correlating with the required function of Arks in cell proliferation events. Biochemical characterization showed the specific and sensitive inhibition of *Pf*Ark1 as the primary target of the inhibitors, with exquisite potency seen for hesperadin and NVP‐TAE684 (TAE684). Hesperadin is a bona fide inhibitor of only *Pf*Ark1, without targeting other kinases or exhibiting additional pleiotropic activity associated with the inhibition of hemozoin formation (the crystalline byproduct of hemoglobin digestion), as is the case with TAE684 and the other kinase inhibitors studied. A unique aspartate residue in the *Pf*Ark1 active site confers selectivity to hesperadin inhibition over the mammalian Aur and *Pf*Ark2, which have a lysine residue at the equivalent position. *Pf*Ark1 is shown to be the main Ark family member involved in mitotic processes, and interference with its activity results in aberrant nuclear division with no clear microtubule nucleation at the CPs in both ABS parasites and male gametes. To our knowledge, this is the first direct chemical evidence of an inhibitor specifically targeting any of the *Pf*Ark members. Notably, we demonstrate that inhibition of *Pf*Ark1 activity can be achieved at single‐digit nanomolar concentrations, with a large selectivity window for the parasite, and a favorable profile for potential drug candidates. These findings expand the current knowledge base regarding kinases as drug targets in malaria parasites and provide chemical validation of *Pf*Ark1 as a druggable target, with hesperadin presenting a starting point for further drug‐discovery initiatives.

## Results and Discussion

### Aur Inhibitors Demonstrate Effectiveness Against the Replicative Stages of *P. falciparum* Parasites

**Figure 1:**
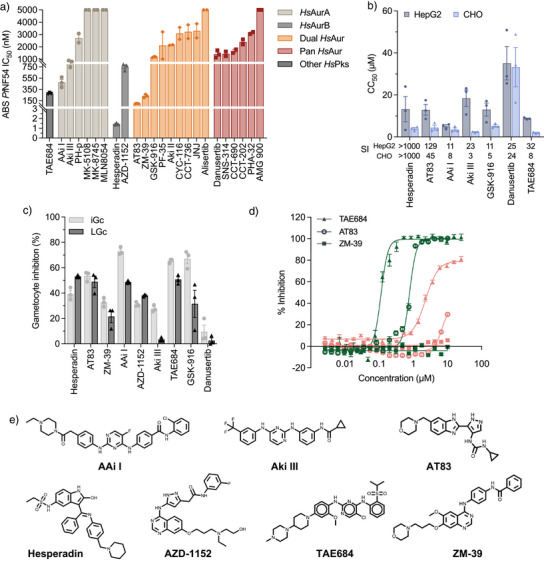
Activity profile of Aur class anticancer inhibitors across the life cycle of *Plasmodium falciparum* parasites. a) Activity (IC_50_) of inhibitors (grouped based on specificity against human Aur) against *P.falciparum* drug‐sensitive (*Pf*NF54) asexual intra‐erythrocytic parasites as measured with SYBR Green I fluorescence as an indicator of viability and proliferation. Values obtained for the most active inhibitors are from three independent biological replicates, each performed in technical triplicate (*n*=3, mean ± S.E.), while the remainder is from two independent biological replicates, each performed in technical triplicate (*n* = 2, mean ± S.E.). b) Cytotoxicity (CC_50_) of selected inhibitors against hepatocellular carcinoma (HepG2) and CHO cell lines. The Selectivity Index (SI = HepG2 CC_50_/*Pf*NF54 IC_50_ and CHO CC_50_/*Pf*NF54 IC_50_) is indicated below the graph (*n* = 3, mean ± S.E.). c) Single point activity profile of selected inhibitors at 5 µM against immature (iGc, II‐III) and late‐stage (LGc, IV/V) gametocytes determined by measuring luminescence of *P. falciparum* parasites expressing luciferase (*n* = 3, mean ± S.E.). d) Activity (IC_50_) against male gamete formation (green) and female gametes (pink) for TAE684, AT83, and ZM‐39 (*n* = 3, mean ± S.E.). e) Structures of the seven most active compounds selected. ABS, asexual blood stages of the parasite; HsAur, human Aur.

A set of commercially available compounds was chosen based on their specificity and potency to the *Hs*Aur members. The inhibitors were first assessed against various life cycle stages of *Plasmodium* parasites in vitro to determine their multistage antiplasmodium activity (Figures 1 and S1). Less than half of the *Hs*AurA inhibitors targeted ABS proliferation of drug‐sensitive NF54 *P. falciparum* parasites with IC_50_ values < 5 µM (Figures 1a and S1). By contrast, both *Hs*AurB, most of the dual‐active inhibitors and five of the six pan‐active Aur inhibitors showed activity at this concentration. Six compounds (AAi I & Aki III [targeting *Hs*AurA^[^ ^28^, ^29^ ^]^], hesperadin & AZD‐1152 [targeting *Hs*AurB^[^ ^30^, ^31^ ^]^], AT83 & ZM‐39 [dual *Hs*AurA&B^[^ ^32^, ^33^ ^]^]) were all potent at < 1 µM. All inhibitors that exhibit specificity towards *Hs*AurB, even when developed as dual *Hs*AurA&B, are generally more potent against *P. falciparum* ABS, with an IC_50_ of 1.5 nM for hesperadin (Figure S2a), and <250 nM for AT83 and ZM‐39. The eight most active compounds retained activity against multidrug‐resistant *Pf*Dd2 and *Pf*K1 strains with ≤ 2‐fold variance in IC_50_ from *Pf*NF54 (Figure S2b). TAE684 (Novartis’ first‐generation anaplastic lymphoma kinase (ALK)‐specific inhibitor) was also included in this assay as it was proposed to target a *Pf*Ark based on Kinobead competitive pulldown data^[^ ^34^ ^]^ (Figure S2c), with TAE684 also presenting potent activity against ABS parasites with an IC_50_ of 280 ± 13 nM.

With selectivity and mammalian cell toxicity often being concerns with kinase inhibitors, we evaluated the activity of the active compounds against hepatocellular carcinoma (HepG2) and Chinese Hamster Ovary (CHO) cell lines (Figure 1b). Several compounds exhibited cytotoxicity with selectivity indices (SI) < 10, including GSK‐916, AAi I, Aki III, and TAE684(CC_50s_ provided in S1)). The pan‐active inhibitor danusertib demonstrated the most pronounced cytotoxic effect against both lines. However, *Hs*AurB inhibitors hesperadin and AZD‐1152 showed distinct selectivity in targeting *P. falciparum* compared to mammalian cell lines, indicating differentiation in action in the parasite, with SI>1000 (CC_50_ of 4.0 ± 1.0 and 13.2 ± 4.9 µM against CHO and HepG2 cells) and >100‐fold (CHO/HepG2 CC_50_ >15 µM), respectively. Similarly, the dual *Hs*AurA&B inhibitors, AT83 (CC_50_ of 4.5 ± 2.1 and CC_50_ of 12.9 ± 4.5 µM, CHO & HepG2) and ZM‐39 (CHO/HepG2 CC_50_ of > 15 µM), also showed SI>100‐fold.

**Figure 2:**
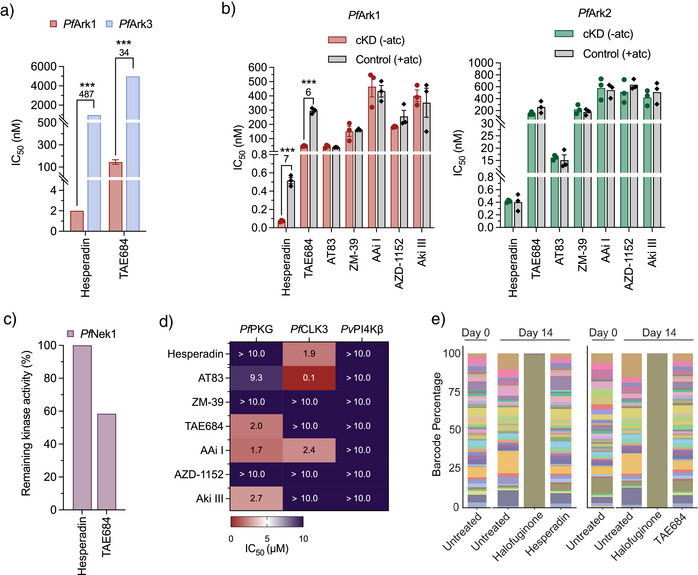
Identification and validation of *Plasmodium* Aurora‐related kinase as the primary target. a) Activity (IC_50_) against recombinant *Pf*Ark1 and *Pf*Ark3 proteins, using a three‐hybrid split‐luciferase competitive binding assay (KinaseSeeker). b) Effect of conditional knockdown (cKD) of *Pf*Ark1 and *Pf*Ark2 on parasite sensitivity relative to control conditions in the presence of high aTc. Representative dose‐response curves are presented for each cKD parasite line (*n*=3, mean ± S.E.) with an unpaired two‐tailed *t*‐test, *p<0.05; **p<0.01; ***p<0.001. c) Single‐point activity profile of selected inhibitors at 1 µM against recombinant *Pf*Nek1 (KinaseSeeker). d) Inhibitory activity against recombinant *Pf*PKG, *Pf*CLK3, and *Pv*PI4Kβ. The mean IC_50_ values ± SD were calculated using two independent experiments (*n* = 2), each with technical duplicates using the ADP‐Glo Kinase Assay. e) Stacked bar plots illustrating barcode populations on days 0 and 14 for no drug, drug control (halofuginone), and selected inhibitors.

We subsequently evaluated the ability of the compounds active on *P. falciparum* NF54 ABS parasites to target additional life‐cycle stages of *P. falciparum* parasites. All selected inhibitors displayed minimal (<50% inhibition at 5 µM) gametocytocidal activity, with only AAi I, GSK‐916, and TAE684 showing ∼70% inhibition of immature gametocyte viability (>80% stage II/III) whilst unable to kill mature gametocytes effectively (Figure 1c). This finding was not unexpected, as gametocytes are non‐proliferative cells, although all three *Pf*Arks are expressed during gametocytogenesis on a transcript and protein level.^[^ ^16^, ^35^ ^]^ Such functional impairment of gametocytes has been described before in other gametocyte‐sterilizing compounds,^[^ ^36^ ^]^ with the effect evident during male gametogenesis. Subsequently, hesperadin (IC_50_ of 10 nM^[^ ^37^ ^]^), AT83 (786 ± 16 nM), and TAE684 (116 ± 17 nM) prevented male gamete exflagellation, whereas only TAE684 had any appreciable activity against female gametes (IC_50_ of 2.3 ± 0.05 µM), with the rest inactive (>10 µM) (Figure 2d). This confirms the ability of these Aur inhibitors to target male gamete formation, which also requires DNA replication and cell division. Hesperadin and TAE684 further display a low nanomolar (IC_50_ <200 nM) potency against *P. berghei* liver schizonts, confirming the preference of these compounds for proliferative forms of the parasite (Figure S2d). Taken together, these data reveal that a focused set of *Hs*Aur‐specific inhibitors demonstrates potent and selective antiplasmodium activity, preferentially targeting proliferative parasite stages related to cell cycle division, while exhibiting an acceptable margin of cytotoxicity. However, in addition to having potential as ABS active in TCP‐1 type strategies,^[^ ^38^ ^]^ the activity against gametes and liver stages raises the possibility of leveraging *Pf*Ark1 inhibition in transmission‐blocking strategies (with both TCP‐3 and TCP‐5 potential).^[^ ^39^ ^]^

### *Pf*Ark1 was Identified and Validated as a Novel *Plasmodium* Kinase Target

Given that the selected inhibitors were developed and optimized to specifically target different *Hs*Aur mitotic kinases, we aimed to correlate the whole‐cell inhibition of the proliferative stages of *P. falciparum* to the biochemical evaluation of the inhibition of *Pf*Ark proteins (Figure 2). We first used the KinaseSeeker competitive binding assay to determine the inhibitory activity against *Pf*Ark1 and *Pf*Ark3.^[^ ^40^ ^]^ Among the selected inhibitors tested, AT83 and AAi I had a marginal effect (AT83: 30% inhibition of *Pf*Ark1, AAi I: 40% inhibition of *Pf*Ark3, Figure S3a). However, hesperadin and TAE684 exhibited potent activity against *Pf*Ark1, with IC_50_ values of 2 ± 0.2 and 146 ± 20 nM, respectively (Figure 2a). Notably, both hesperadin and TAE684 had a significant preference towards *Pf*Ark1, with hesperadin ∼480‐fold more active against *Pf*Ark1 than *Pf*Ark3 (*p *= 0.0004 and *p = *0.0001, respectively, *n* = 3, unpaired Student's *t*‐test). Moreover, the 2 nM activity of hesperidin on *Pf*Ark1 protein correlates with the in vitro activity against drug‐sensitive *Pf*NF54 parasites at 1.5 nM.

We evaluated the involvement of *Pf*Ark2 inhibition by determining the loss of activity of the compounds against conditional knockdown (cKD) lines of *P. falciparum* for either *Pf*Ark1 or *Pf*Ark2.^[^ ^41^ ^]^ Most compounds did not show a change in activity against the cKD of either *Pf*Ark1 or *Pf*Ark2 (Figure 2b). However, cKD of *Pf*Ark1 resulted in an increased sensitivity to hesperadin and TAE684, as evidenced by a significant > 5‐fold decrease in the IC_50_ values compared to the wild‐type control (*p* = 0.0002 *and p* = 0.00003, respectively, *n* = 3, unpaired Student's *t*‐test; Figures 2b and S3b), associated with decreased protein levels of *Pf*Ark1 under cKD conditions. Conversely, there was no change in the IC_50_ value for these compounds in the *Pf*Ark2 cKD. This further supports *Pf*Ark1 as the primary protein target for hesperadin and TAE684 (Figure 2c).

The specificity towards *Pf*Ark1 was confirmed by evaluating the ability of the compounds to inhibit other currently relevant antimalarial Ser/Thr or lipid kinase drug targets. This included inhibition of *Pf*Nek1, as hesperadin resistance selections previously yielded mutations in this gene, suggesting an epistatic interaction between *Pf*Ark1 and *Pf*Nek1,^[^ ^27^ ^]^ and *P. berghei* Nek1 exhibits similar spatiotemporal associations with the outer CP domain as *Pf*Ark1.^[^ ^42^ ^]^ However, hesperadin did not inhibit *Pf*Nek1 activity, even at 1 µM, with only TAE684 showing a marginal 40% effect on this protein (Figure 2c). The inhibitors did not exhibit noteworthy activity against three other validated antimalarial kinase targets (*Pf*PKG, *Pf*CLK3, or *Pv*PI4Kβ) (Figure 2d), except for AT83, which inhibited *Pf*CLK3 with an IC_50_ of 100 nM. However, it is important to note that the kinase assay was performed at a low ATP concentration (10 µM). According to the Cheng‐Prusoff equation, the *Pf*CLK3 IC_50_ will be ∼100‐fold higher in the presence of cellular ATP concentrations (∼3 mM in *P. falciparum* parasites). Thus, inhibition of *Pf*CLK3 within the parasite is predicted to be weak and unlikely to contribute substantially to the observed antiplasmodial activity.

Additionally, hesperadin, TAE684, and AT83 were evaluated for their ability to inhibit the proliferation of a set of resistant *P. falciparum* parasites (including resistance mutants for *Pf*PI4k and *Pf*CLK3) using the antimalarial resistome barcode sequencing (AReBar) assay.^[^ ^43^ ^]^ All three compounds killed all of the resistant lines in the platform (Figures 2e, S3c, and S2), indicating no cross‐resistance with known antimalarial resistance mechanisms and a novel mode of action.

These data indicate that the specific inhibition of *Pf*Ark1 within the family of Arks in *P. falciparum* by hesperadin and TAE684 is the primary driver of ABS antiplasmodium activity. This correlates with the specific increase in abundance of this protein (over *Pf*Ark2) peaking at ∼28–34 hpi^[^ ^44^ ^]^ in preparation for its availability during schizogony (Figure S3d).

The validation of *Pf*Ark1 as a druggable target expands and diversifies the compendium of targetable mitotic kinases in *Plasmodium* beyond the current indication of *Pf*Nek3 inhibition with BI‐2536, a known potent human polo‐like kinase 1 inhibitor.^[^ ^45^ ^]^ This provides a clear starting point for drug repurposing and repositioning strategies against malaria. Hesperadin is also active against *T. brucei* and *Leishmania major*, with initial hit expansion indicating that analogues mirror hesperadin's activity and phenotype.^[^ ^24^ ^]^ This provides support for structure‐activity relationship (SAR) expansion studies and further development of hesperadin as an antiplasmodium chemotype. Initial data on the in vitro ADME properties of the frontrunner compounds (hesperadin, TAE684, and AT83) indicate metabolic liabilities for hesperadin, particularly in human liver microsomes, whereas AT83 displays a favorable half‐life of 395 min in microsomes, but with potential phase II liabilities as indicated by low stability in rat hepatocytes (Table 1). These data provide baseline information to guide design and optimization of a next generation of derivatives in hit‐2‐lead optimization campaigns to progress these compounds.

**Table 1:** Summary of in vitro ADME properties of selected active compounds.

			HL microsome^a)^		Rat hepatocyte				
	eLogD pH 7.4	Kin sol (µM) pH 7.4	CLint (µL min^−1^ mg^−1^)	t1/2 (min)	CLint (µL min^−1^/10e6 cells)	*t*1/2 (min)	Protein binding (%)^b^	tPSA	MW (Da)
**AT83**	2.2	22.2	3.5	395	12.6	55	16.5	111	381
**TAE684**	3.4	198.5	25.4	55	3.5	197	80	103	614
**Hesperadin**	2.8	2.5	76.1	18	14.2	49	37.3	98	517

^a)^ Human liver microsomes.

^b)^Protein binding as measured in Albumax II media.

### The Activity of the Additional *Hs*Aur Inhibitors is not Associated with Ark Inhibition

**Figure 3:**
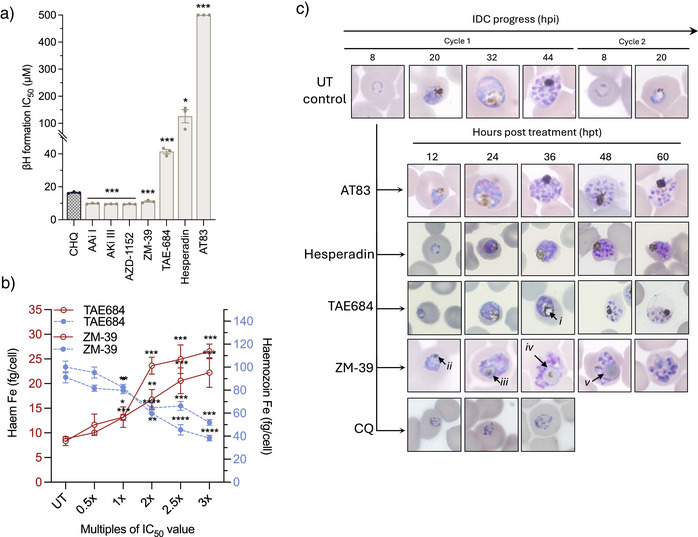
Phenotypic effect of potent *Hs*Aur inhibitors on intra‐erythrocytic development. a) Measuring the ability of the inhibitors to interfere with the formation of synthetic hemozoin, βH, in vitro in a cell‐free detergent‐mediated NP‐40 assay. Error bars represent ± SD for technical triplicates with an unpaired two‐tailed Student's *t*‐test. Exact *p*‐values provided in *p<0.05; **p<0.01, ***p<0.001, ****p<0.0001. b) Dose‐dependent changes in the heme Fe levels from intracellularly‐extracted fractions of hemozoin under ZM‐39 and TAE684 treatment. c) Phenotypic response of ABS *Pf*NF54 parasites exposed to AT83 (3xIC_50_, ∼600 nM), ZM‐39 (3xIC_50_, ∼750 nM), TAE684 (3xIC_50_, ∼900 nM) and hesperadin (IC_99_, ∼3 µM). Parasite morphology was observed at 12 h intervals using thin blood smears and indicated enlarged food vacuoles (*i*, *iv*, and *v*), and small hemozoin crystals (*ii* and *iii*).

Since all the compounds selected for this study were based on their activity against *Hs*Aur, but we could only convincingly show that hesperadin and TAE684 target *Pf*Arks, we explored alternative mechanisms of activity for some of the most potent inhibitors (AT83, ZM‐39, Aki I, AAi III, and AZD‐1152). Several kinase inhibitors inhibit hemozoin formation due to structural similarities in the presence of multiple heteroaromatic rings, planar structures, and basic centers.^[^ ^46^ ^]^ Evaluation of the ability to block formation of synthetic β‐hematin (βH) in vitro in a cell‐free detergent‐mediated Nonidet P‐40 (NP‐40) assay^[^ ^47^ ^]^ indicated that AAi I, Aki III, AZD‐1152, and ZM‐39 (but not AT83) were potent inhibitors of βH formation (IC_50 _< 20 µM), similar to the positive control chloroquine (CQ) (Figure 3a). This could implicate inhibition of hemozoin formation as a primary mode of action for these compounds. Interestingly, TAE684 displayed some effect against βH formation (IC_50_ of 41.4 ± 2.3 µM, Figure 3a), whereas hesperadin was ∼3‐fold less active (IC_50_ of 126.3 ± 42.0 µM). The inhibition of βH formation translated to inhibition of intracellular hemozoin formation for both ZM‐39 and TAE684,^[^ ^48^ ^]^ which caused a significant increase in free heme (*p = *0.00000002 and *p = *0.0003, respectively, *n* = 3, unpaired Student's *t*‐test), accompanied by a simultaneous decrease in hemozoin formation (*p = *0.00001 and *p = *0.00000003, respectively, *n* = 3, unpaired Student's *t*‐test) (Figure 3b). This effect was not observed in the hesperadin treatment, with no change in either heme or hemozoin levels, implying that hesperadin does not affect hemozoin formation in the parasite (Figure S4a).

This was confirmed by a distinct phenotypic morphology, where a decreased hemozoin crystal size (Figure 3c–i,ii) and an enlarged food vacuole‐like structure (*iii* & i*v*) were observed for ZM‐39 treatment within the first 24 h post‐treatment (hpt). Importantly, the TAE684 treatment had a distinctly different phenotype, whereas hemozoin formation persisted for the first 24 h, but thereafter, a distinct vacuolar structure was present, and aberrant schizonts were formed. The morphological abnormalities associated with the food vacuole were not present in either hesperadin‐ or AT83‐treated parasites. For both situations, parasites normally progressed and entered schizogony; however, schizonts were abnormal, particularly following hesperadin treatment, and persisted for 60 hpt, indicating an arrested state and functional impairment in the completion of schizogony (Figures 3c and S4b). Co‐treatment of TAE684 or ZM‐39 with CQ, a known hemozoin formation inhibitor, showed an additive or indifferent effect, as evaluated by fixed‐ratio isobologram analysis, with ΣFIC_50_ values of 1.3 and 1.4, respectively. By contrast, hesperadin was antagonistic to CQ (ΣFIC_50_ 1.6) as well as to TAE684 (ΣFIC_50_ 3.5) (Figure S5). Taken together, the data suggest that TAE684 exhibits polypharmacology, involving both the inhibition of hemozoin formation and *Pf*Ark1 inhibition, similar to other kinase inhibitors,^[^ ^49^ ^]^ but importantly, hesperadin has *Pf*Ark1 as its singular target.

### In Silico Investigation of Inhibitor–Target Interactions in the ATP‐Binding Site of *Pf*Ark1

**Figure 4:**
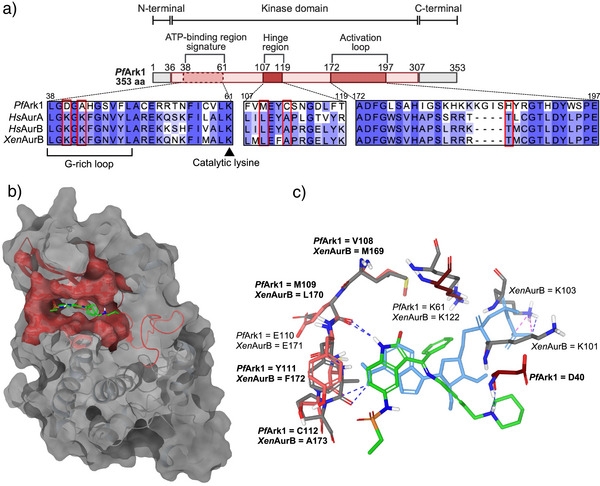
In silico modeling predicts protein‐inhibitor interactions in the active site of *Pf*Ark‐1. a) Diagram of key characteristics of *Pf*Ark1 protein along with a protein sequence alignment of mammalian and *Plasmodium falciparum* Aur. b,c) Hesperadin binding pose in *Pf*Ark1 (green), overlayed with the binding pose of ATP (light blue), and the *Xenopus laevis* AurB crystal structure (2BFY, gray), with residue differences indicated in bold. The PfArk1 homology model was derived from the crystal structure of human Aur A (HsAurA) co‐crystallized with ATP (PDB 5DNR), with a root mean square deviation (RMSD) of 0.44Å and no stereochemical violations. Selected main chains and side chains of key residues conserved within the kinase domain are displayed (red and pink are for *Pf*Ark1, while gray is for *Xen*AurB) with hydrogen bonds displayed as blue and salt bridge bonds as pink dashed lines.

The specificity of hesperadin, an ATP‐competitive inhibitor, against *Pf*Ark1 was mechanistically investigated. *Pf*Ark1 shares ∼34% identity with mammalian AurA and B, with both the ATP‐binding signature and S/T PKs sites well‐conserved, including the catalytic lysine residue, Lys61 (Figures 4a and S5A). However, *Pf*Ark1 exhibits several critical changes in both the ATP binding site and the active site relative to both mammalian and protozoan Aur proteins, including alterations of two conserved Lys residues, one to Asp (at position 40) and Ala (at position 42), something only seen for the *T. gondii* Ark1, but not for *Pf*Ark2 (Figure S5b). Additionally, there are changes in the gatekeeper residue from Leu to a bulkier and more flexible Met at position 109 and an Ala to Cys change at position 112 within the hinge region (Figure 4a).

In silico molecular docking studies revealed that hesperadin binds within the ATP‐binding pocket of *Pf*Ark1 but in a different pose to that observed for *Hs*AurB and in the *Xenopus laevis* AurB co‐crystallized with hesperadin^[^ ^50^ ^]^ (Figure 4b,c). Hesperadin's indolinone moiety and sulfonamide group form hydrogen bonds, respectively, with the key conserved hinge region residues Glu110 and Tyr111. This causes the central phenyl to point into the active pocket of *Pf*Ark1, which would displace the α‐phosphate of ATP and prevent its interaction with the catalytic lysine (Lys61) (Figure 4c). Indeed, analogues lacking the bulky phenyl are not as active as hesperadin.^[^ ^24^ ^]^ Hesperadin is additionally stabilized in the ATP binding site pocket by an H‐bond between its piperidine ring and the unique Asp40 found in the *Plasmodium* enzyme (Figure 4c). *Pf*Ark1 is differentiated from *Pf*Ark2 in this position, with the latter containing a more conserved K–N modification compared to the *Hs*Aur, which does not accommodate stabilization of hesperadin to the same extent as in *Pf*Ark1. This data provides clarity on the selectivity of hesperadin for *Pf*Ark1 over both *Pf*Ark2 and mammalian AurA and B. Moreover, the unique Cys residue in the hinge region of *Pf*Ark1 is additionally particularly interesting from a drug development point of view as it undergoes unique interactions with the inhibitor, which could be exploited in the design of potential covalent inhibitors as highly potent and selective inhibitors, as has been shown for inhibition of *Pf*CLK3 kinase.^[^ ^51^ ^]^

### Inhibition of *Pf*Ark1 Affects the Progression of Schizogony

**Figure 5:**
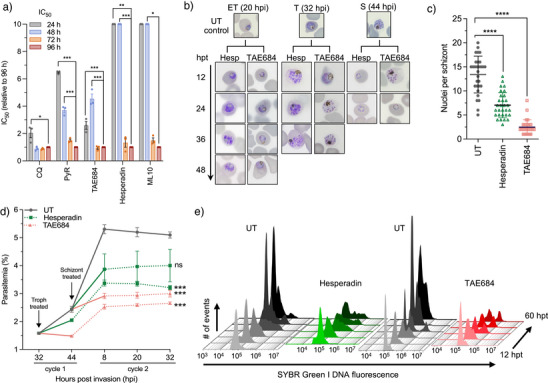
Effect of *Pf*Ark1 inhibition on intra‐erythrocytic development. a) IC_50_ speed of kill assay using unsynchronized *Pf*NF54. CQ, pyrimethamine (PyR). b) Morphological evaluation (Giemsa‐stained thin smears) of *P. falciparum* asexual development following treatment with TAE684 (3xIC_50_, ∼900 nM) and hesperadin (IC_99_, ∼3 µM) (hpt – hours post‐treatment; hpi – hours post‐invasion*)*. c) Nuclei count per schizont after treatment (*n*=30). Error bars represent the 95% confidence interval (CI) of the mean. d) Synchronized *Pf*NF54 mature trophozoite and schizont populations treated with TAE684 and hesperadin for a 12 h period (solid line), washed off, and parasitemia measured at 12 h intervals using flow cytometry. e) Flow cytometric analysis of nuclear division following hesperadin and TAE684 treatment, sampled 12 hpt, and each subsequent 12 h until 60 hpt. Nuclei content was detected by consecutive staining with SYBR Green I (DNA fluorescence), detected in the FITC channel. Histograms overlaid for a representative sample of biological triplicates. All data are from three independent biological replicates, each performed in technical duplicates (*n *= 3, mean ± S.E.), significance was calculated using a two‐tailed Student's *t*‐test, *p<0.05, ***p<0.001, ****p<0.0001.

We subsequently evaluated the effects of hesperadin and TAE684 against the proliferative stages of the parasites and their specificity for *Pf*Ark1. The rate at which hesperadin and TAE684 kill the parasite was evaluated by determining shifts in IC_50_ over time.^[^ ^52^ ^]^ Hesperadin and TAE684 kill kinetics indicate that their effect is only evident with a significant IC_50_ shift after 48 h, indicative of activity within one life cycle (Figure 5a). This profile resembles that seen for the *Pf*PKG inhibitor ML10, which prevents parasite egress and invasion, but not for fast‐acting compounds such as CQ (Figure 5a). To further evaluate which developmental stage during asexual proliferation is affected by hesperadin and TAE684 treatment, we treated tightly synchronized parasites at 12 h intervals, correlating to ring, early, and late trophozoites and schizonts (Figure 5b). Hesperadin treatment had minimal impact on rings or trophozoites and did not limit the maturation from rings to trophozoites, whereas TAE684 treatment consistently affected trophozoite food vacuole formation (Figures 5b and 3c). The most pronounced effect for both compounds was associated with the completion of schizogony at ∼36–44 hpi, with no progression into the next cycle. Further evaluation of the morphologically aberrant schizonts revealed a significant reduction in the number of daughter merozoites formed for both hesperadin and TAE684 treatment (*p *< 0.000001, *n* = 30, unpaired Student's t‐test), an effect that was pronounced for the comparative treatment with TAE684 (Figure 5c). Hesperadin treatment did not prevent already formed schizonts from invading and forming rings in the next cycle, a process that was somewhat delayed by TAE684. Although this phenotype is reminiscent of the effect of *Pf*PKG inhibition, which also prevents parasite egress and subsequent invasion,^[^ ^53^ ^]^ the lack of *Pf*PKG inhibition for hesperadin and TAE684 and their indifferent effect when combined with ML10 (ΣFIC_50_ of 1.1 and 1.3, respectively, Figure S6) support a *Pf*PKG‐independent mechanism for these compounds. However, the data delineate a timeframe of action of hesperadin associated with the completion of late schizogony processes.

Flow cytometric quantification of this effect was performed after a 12 h drug treatment pulse on trophozoite and early schizont populations before drug washout (Figure 5d). Both hesperadin and TAE684‐treated trophozoites (∼30 hpi) were able to recover from a 12 h pulse and progress to schizonts; however, these parasites were unable to sufficiently establish reinvasion, resulting in a significant (*p *= 0.00015 and *p *= 0.00004, respectively, *n* = 3, unpaired Student's t‐test) decrease in parasitemia in the subsequent population. A similar significant effect was immediately evident with TAE684‐treated schizonts (*p *= 0.0002, *n* = 3, unpaired Student's *t*‐test) (∼40–42 hpi); however, no significant effect was observed for hesperadin‐treated schizonts (*p *= 0.139, *n* = 3, unpaired Student's *t*‐test), reminiscent of the phenotype observed in schizont‐treated samples (Figure 5b,d). Quantification of the nuclear content of treated parasites indicated entry into schizogony compared to untreated populations, but with the treatment primarily affecting parasites during schizogony, and the fraction of individual cells halted in the schizont stage containing ≥ 4n (DNA content) was higher in drug‐treated compared to untreated parasites (Figure 5e). Taken together, hesperadin as a *Pf*Ark1 inhibitor primarily affects parasite progression through schizogony (mid‐to‐late schizont development), where cells are unable to complete nuclear division and segregation successfully. However, it is ineffective against mature schizont stages, where these processes have already been completed.

### *Pf*Ark1 is Critical to the Completion of Mitotic Processes

**Figure 6:**
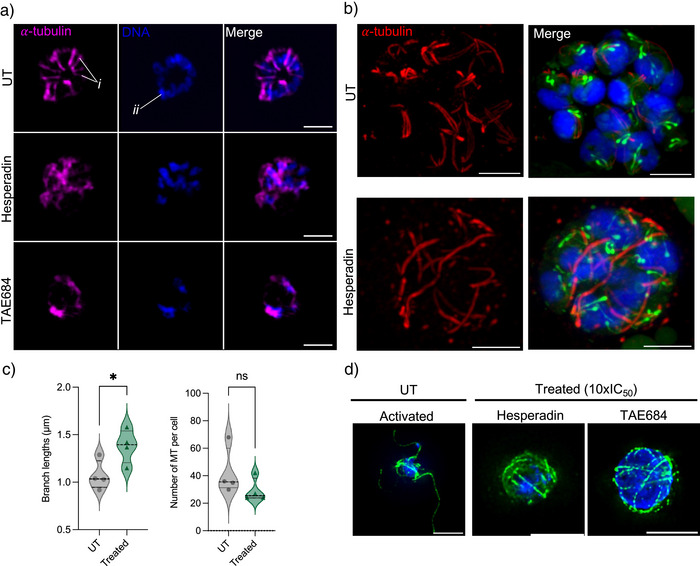
*Pf*Ark1 inhibition effect on microtubule and nuclear material morphology. Ring stage, synchronized, asexual intra‐erythrocytic *Pf*NF54 parasites treated with hesperadin and TAE684 were harvested at ∼46 hpi. a) Representative images (maximum intensity projections) of the morphological abnormalities observed in nuclei (Hoechst, blue) & microtubules (anti‐⍺‐tubulin, pink), showing subpellicular microtubules (SPMTs, *i*), nuclei (*ii*). The images represent at least ten parasites per sample. Scale bars correspond to 2 µm. b) Representative images (maximum intensity projections) of expansion microscopy images of UT mature schizonts (top panel) compared to hesperadin‐treated (bottom panel). Red = tubulin, blue = DNA, and green = NHS‐ester. Scale bars correspond to 5 µm. c) Average microtubule (MT) branch lengths and number of MT branches per cell of UT compared to hesperadin‐treated, *n* = 4 schizonts, mean ± SD with an unpaired Welch's two‐tailed *t*‐test. d) Representative images of TAE684 and hesperadin‐treated *Plasmodium falciparum* male gametes labeled with anti‐⍺‐tubulin (green) and co‐stained with DAPI for nuclei (blue). Scale bars correspond to 5 µm.

Fluorescence microscopy morphological evaluation of ABS parasites, where *Pf*Ark1 activity was inhibited by hesperadin or TAE684, revealed nuclear morphological abnormalities in schizonts. The nuclei of those treated with hesperadin appeared distinctly multi‐lobed, whereas TAE684‐treated schizonts had more elongated nuclei (Figure 6a), although packaging into daughter cell structures did occur, as evident by nuclear membrane detection (Figure S7a). The abnormal shape and decreased number observed during nuclear division were associated with abnormalities in MT structures in hesperadin‐ and TAE684‐treated schizonts. Compared to the well‐defined SPMTs seen in untreated schizonts, hesperadin treatment resulted in disorganized and interconnected MT structures. TAE684 decreased the appearance of MT structures entirely, with an overall decrease in nuclear content (Figure 6a).

Expansion microscopy (ExM) was subsequently used to provide more nuanced evaluation of hesperadin‐treated cells, which revealed the extent of abrogation of nuclear segmentation and packaging, as well as extensive MT defects (Figure 6b). Untreated parasites contained the expected well‐packaged and segregated daughter nuclei, with SPMTs clearly extending from the MTOCs associated with the CPs in proximity to well‐formed rhoptries (Figure 6b). No intranuclear MTs were evident as segregation was completed. By contrast, hesperadin treatment caused parasites to halt in schizogony at a point where the parasites contained multilobed nuclei, with the nuclear material not separated in most instances, and nuclei showing an increased nuclear volume. The MT organization was clearly affected by hesperadin treatment, with significantly extended MT structures (average lengths of 1.4 ± 0.7 µm, versus untreated parasites at 1.1 ± 0.4 µm, *p* = 0.04, *n* = 4, Figure 6c) spanning across and connecting different nuclear centers/CPs. Fewer of these MT structures were present per cell (29 versus 42 in hesperadin versus UT cells), and fewer could be associated with MTOC and the limited rhoptry pairs formed. This suggests that these microtubule structures are halted as interpolar MTs that are unable to retract during the interpolar to hemi‐spindle transition, as is typically required.^[^ ^54^, ^55^ ^]^ This suggests that *Pf*Ark1 function is necessary for meta‐ and anaphase‐like transition processes during mitosis in the parasite. However, the potential for these aberrant MTs to be malformed, extended SPMTs cannot currently be excluded. Indeed, some of these aberrant MT structures closely localize to rhoptries connected to associated apical polar rings, as one would expect from SPMTs (Figure 6b).

We subsequently evaluated the broader involvement of *Pf*Ark1 in mitotic processes also present during male gametogenesis. Under normal conditions, when mature male gametocytes are successfully activated, exflagellation results in the formation of eight MT‐labeled flagella, each associated with segregated nuclei (Figure 6d) compared to non‐activated mature gametocytes (Figure S7b). Treatment with TAE684 and hesperadin was associated with some DNA replication proceeding normally, but the nuclear material remained highly compacted and lacked proper segregation. Strikingly, the MT structures in the drug‐treated cells are abnormal, thin, and wrapped around the nucleus, pointing to disorganized MT forming flagellae (Figure 6d). This suggests that DNA replication has mostly occurred, but the absence of *Pf*Ark1‐mediated signaling disrupted microtubule organization on the basal body in these stages. The extended, thin phenotype closely resembled that observed during late schizogony under *Pf*Ark1 inhibition (Figure 6b). These findings indicate that *Pf*Ark1 activity is required post‐DNA replication but before nuclear segregation and egress of male gametes, suggesting a mitotic block during gametogenesis.^[^ ^37^ ^]^ These findings suggest that the disruption of *Pf*Ark1 function during male gametogenesis does not affect DNA replication but rather may disrupt proper kinetochore attachment and spindle formation.

Our high‐resolution imaging provides the first evidence that disruption of *Pf*Ark1 function causes complete disorganization of MT structures required to coordinate and complete mitosis. The multilobed nuclei observed are reminiscent of the inhibition of AurB (equatorial Aur) in other organisms and apicomplexan parasites, which results in misaligned chromosomes, lagging chromatids, cytokinesis failure, and polyploidy.^[^ ^26^, ^30^, ^56^ ^]^ Our observations support *Pf*Ark1 to be functionally similar to AurB in its association with kinetochores until metaphase, and the extended MT structures after loss of *Pf*Ark1 function imply a lack of translocation to the central spindle to coordinate cytokinesis. Although the disruption of AurB function can lead to disruption of microtubule dynamics and structure, these effects are also observed in the inhibition of AurA, which leads to unaligned chromosomes due to impaired centrosome separation and the formation of monopolar spindles, which could explain the spindle structures we observe in mature treated schizonts.^[^ ^19^, ^57^ ^]^ Thus, based on these phenotypic observations, we suggest that *Pf*Ark1 fulfills the mitotic responsibilities of both AurA and AurB to control correct mitotic and interpolar spindle formation in ABS parasites and male gametocytes. Co‐localization with known markers of the centrosome (e.g., Centrins^[^ ^58^ ^]^), kinetochores (e.g., NDC80^[^ ^59^ ^]^), and chromosome passenger proteins (e.g., INCENP) will be needed to confirm if *Pf*Ark1 takes on a role of functional redundancy in *P. falciparum* for both AurA and B.

## Conclusion

We demonstrate that *Pf*Ark1 is the most vulnerable target from the Ark protein family in *P. falciparum*, correlating with its essential requirement across multiple life cycle stages of the parasite.^[^ ^10^ ^]^ Moreover, *Pf*Ark1 inhibition abrogates mitotic‐associated proliferation processes, suggesting that *Pf*Ark1 is the primary Aur member governing mitotic‐related processes^[^ ^14^ ^]^ in ABS parasites, male gametes,^[^ ^60^ ^]^ and most likely also during hepatocyte schizogony. *Pf*Ark1 inhibition results in abnormal nuclear morphology, along with spindle structure defects that we previously described.^[^ ^27^ ^]^ With hesperadin as a tool compound that exclusively inhibits *Pf*Ark1, we could deduce the functional and mechanistic importance of this protein during mitosis. We propose that *Pf*Ark1 is the most important Aur regulating mitotic processes in the parasite and that hesperadin, as a potent and selective inhibitor of *Pf*Ark1 in *P. falciparum*, can be considered for future development as an antimalarial.

## Supporting Information

The authors have cited additional references within the Supporting Information.^[^ ^1^, ^2^, ^3^, ^4^, ^5^, ^6^, ^7^, ^8^, ^9^, ^10^, ^11^, ^12^, ^13^, ^14^, ^15^, ^16^, ^17^, ^18^, ^19^, ^20^, ^21^, ^22^, ^23^, ^24^, ^25^, ^26^, ^27^, ^28^, ^29^, ^30^, ^31^, ^32^, ^33^, ^34^, ^35^, ^36^ ^]^

Supplementary File with Supplementary Figures S1–S7 and Supplementary Methods.

Supplementary data files S1, S2.

## Author Contributions

HL performed the work with MM, T Rabie, and JT (modeling and docking). HL and KM performed the hematin experiments. KM performed the hemozoin experiments under KJW, with NS performing recombinant protein work under LBC with KC. SGD, LCG, NB, MTF, MLdS, and JF contributed experimental data with interpretations and validations under JCN, MSL, MD, and EAW. LMB conceptualized the study and wrote the paper with HL. All co‐authors contributed to and approved the final version of the manuscript.

## Conflict of Interests

The authors declare no conflict of interest.

## Supporting information



Supporting Information

Supporting Information

Supporting Information

## Data Availability

The data that support the findings of this study are available in the Supporting Information of this article.
