# Supplementary material for: Targeting Aurora Kinases as Essential Cell‐Cycle Regulators to Deliver Multi‐Stage Antimalarials Against Plasmodium Falciparum
Source: Angew Chem Int Ed Engl. 2025 Oct 23;64(51):e202518493. doi: 10.1002/anie.202518493 (PMC12707362; doi:10.1002/anie.202518493)
Supplement: Supplementary file 1 — Supporting Information [file ANIE-64-e202518493-s003.docx]

SUPPORTING INFORMATION

Targeting Aurora Kinases as Essential Cell-cycle Regulators to Deliver Multi-stage Antimalarials against *Plasmodium falciparum*

Henrico Langeveld^[1],[2]^, Keletso Maepa^[3],[4]^, Marché Maree^[1],[2]^, Jessica L. Thibaud^[5]^, Nicolaas Salomane^[3],[4]^, Rosie Bridgwater^[6],[7]^, Mufuliat T. Famodimu^[6],[7]^, Luiz C. Godoy^[8]^, Charisse Flerida A. Pasaje^[8]^, Nonlawat Boonyalai^[9]^, Mariana Laureano de Souza^[10]^, Justin Fong^[10]^, Tayla Rabie^[1],[2]^, Mariëtte van der Watt^[2]^, Rensu P. Theart^[11]^, Sonja Ghidelli-Disse^[12]^, Jacquin C. Niles^[8]^, Marcus C. S. Lee^[9]^, Elizabeth A. Winzeler^[10]^, Michael J. Delves^[6],[7]^, Kelly Chibale^[3],[4]^, Kathryn J. Wicht^[3],[4]^, Lauren B. Coulson^[3],[4]^, Lyn-Marié Birkholtz^[1],[2],[5]*^

^[1]^ Department of Biochemistry, Genetics and Microbiology,^[2]^ Institute for Sustainable Malaria Control, University of Pretoria, Hatfield, Pretoria, 0028, South Africa

^[3]^South African Medical Research Council Drug Discovery and Development Research Unit, Department of Chemistry and Institute of Infectious Disease and Molecular Medicine, University of Cape Town, Rondebosch 7701, South Africa

^[4]^Holistic Drug Discovery and Development (H3D) Centre, University of Cape Town, Rondebosch 7701, South Africa

^[5]^Department of Biochemistry, Stellenbosch University, Stellenbosch, Matieland, 7602, South Africa

^[6]^LSHTM Malaria Centre, London School of Hygiene and Tropical Medicine, London, UK

^[7]^Department of Infection Biology, Faculty of Infectious Tropical Diseases, London School of Hygiene and Tropical Medicine, London, UK

^[8]^Department of Biological Engineering, Massachusetts Institute of Technology, Cambridge, MA 02139, USA

^[9]^Division of Biological Chemistry and Drug Discovery, Wellcome Centre for Anti-Infectives Research, University of Dundee, Dundee, UK

^[10]^Department of Pediatrics, School of Medicine, University of California, San Diego, La Jolla, CA 92093, USA

^[11]^Department of Electrical and Electronic Engineering, Stellenbosch University, Stellenbosch, Matieland, 7602, South Africa

^[12]^Cellzome GmbH, a GSK Company, Heidelberg, Germany

***Corresponding author:** Lyn-Marié Birkholtz; Orcid.org/0000-0001-5888-2905; [lbirkholtz@sun.ac.za](mailto:lbirkholtz@sun.ac.za)

Additional supporting information can be found online in the Supporting Information section

**CONTENTS**

1. **Supplementary Figures**

**Supplementary Figure S1** – Structures of the selected aurora kinase class anticancer inhibitors

**Supplementary Figure S2** – **(A)** Hesperadin dose response curve, **(B)** activity against *P. falciparum* multidrug-resistant drug-resistant parasite lines, **(C)** Competitive inhibition data from Kinobeads assay for TAE684 and **(D)** *in vitro P. berghei* ANKA liver stage assay.

**Supplementary Figure S3** – **(A)** Biochemical *Plasmodium* kinase activities, **(B)** Dose-response curves of conditional knockdown (cKD) Ark1, **(C)** AReBar cross-resistance growth curves, **(D)** *P. falciparum* Ark gene expression profiles.

**Supplementary Figure S4** – **(A)** Hesperadin cellular heme fractionation data, **(B)** MitoTracker viability data, **(C)** Fixed-ratio isobologram curves with CQ

**Supplementary Figure S5** – **(A)** Schematic representation of *P. falciparum* Ark1 and -2 and sequence alignment, **(B)** Sequence alignment of other eukaryotic organisms and *P. falciparum* aurora kinase proteins.

**Supplementary Figure S6** – Fixed-ratio isobologram aur inhibitors with ML10

**Supplementary Figure S7** – **(A)** BODIPY-membrane fluorescent images, **(B)** Non-activated mature gametocyte

1. **Supplementary Methods**
   1. Parasite *in vitro* cultivation
   2. *In vitro* asexual and gametocyte activity evaluation
   3. *P. berghei* (*Pb*Luc) liver stage screening
   4. *P. falciparum* Dual Gamete Formation Assay (*Pf*DGFA)
   5. Cytotoxicity counter-screening
   6. Rate- and stage-specific evaluations and inhibitor reversibility
   7. Inhibitor susceptibility assays using *P. falciparum* kinase cKD lines
   8. Antimalarial resistome barcode sequencing (AReBar) cross-resistance assay
   9. *Pf*PKG, *Pv*PI4Kβ and *Pf*CLK3 Kinase Assays
   10. Molecular docking
   11. Isobologram analysis
   12. NP-40 mediated cell-free β-hematin and hemozoin inhibition assays
   13. Fluorescence and U-ExM microscopy
   14. Statistical analysis
   15. In vitro ADME
2. **Supplementary Figures**

**Fig. S1**

**Supplementary Figure S1:** Structures of the selected aurora kinase class anticancer inhibitors, classified according to their human aurora kinase inhibitor class.

**Fig S2.**

**Supplementary Figure S2: (A)** Dose-response curve of Hesperadin against ABS *Pf*NF54, *Pf*Dd2 and *Pf*K1. **(B)** Antiplasmodial activity of nine anticancer aurora kinase inhibitors (selected based on their *Pf*NF54 ABS activity) against *P. falciparum* multidrug-resistant ABS parasites (*Pf*Dd2 and *Pf*K1). **(C)** Kinobead competitive inhibition data. A total of three *Plasmodium* kinases were competed from Kinobeads by TAE684 at 10 µM with a log_2_ fold change below the cutoff of -1 relative to the vehicle control. **(D)** Prophylactic antiplasmodial activity (IC_50_) of Hesperadin and TAE684 in an *in vitro P. berghei* ANKA liver stage assay (n=3, mean ± S.E.).

**Fig S3.**

**Supplementary Figure S3:** **(A)** Single-point activity profile of selected inhibitors at 1 µM against recombinant *Pf*Ark-1 and *Pf*Ark-3 proteins, utilising a three-hybrid split-luciferase competitive binding assay (KinaseSeeker™). **(B)** Effect of conditional knockdown (cKD) of *Pf*Ark-1 on parasite sensitivity to Hesperadin and TAE684, relative to control conditions in the presence of high aTc. Representative dose-response curves are presented for each cKD parasite line (n=3, mean ± S.E.) with an unpaired two-tailed t-test. **(C**) Cumulative growth profiles for drug-treated and no-drug controls. **(D)** Expression profile of *Pf*Ark members across ABS, gametocytogenesis, as well as during G_1_/S-like arrest.

**Fig S4.**

**Supplementary Figure S4:** **(A)** Dose-dependent changes in the heme Fe levels from intracellularly-extracted fractions of hemozoin under hesperadin treatment. **(B)** MitoTracker viability of treated parasites 48 and 60 hpt. Scale bar = 5 µm. **(C)** Fixed-ratio isobologram analysis for selected inhibitors in various combinations against asexual intra-erythrocytic parasites. Data represent the mean fractional inhibitory concentration (FIC_50_) of two biological replicates; each performed in technical duplicates. Error bars represent ± S.E.

**Fig S5.**

**Supplementary Figure S5: (A)** Schematic representation of *P. falciparum* Ark1 and -2 and sequence alignment of conserved motifs of mammalian aurora kinases. **(B)** Sequence alignment of other eukaryotic organisms and *P. falciparum* aurora kinase proteins. Identity/similarity is indicated with the blue shading, and red boxes indicate critical changes in the *Plasmodium* protein.

**Fig S6.**

**Supplementary Figure S6:** Fixed-ratio isobologram analysis for selected inhibitors in various combinations against asexual intra-erythrocytic parasites. Data represent the mean fractional inhibitory concentration (FIC_50_) of two biological replicates; each performed in technical duplicates. Error bars represent ± S.E.

**Fig S7.**

**Supplementary Figure S7:** **(A)** Representative images (average intensity projections) of the morphological effect observed on membrane formation in hesperadin and TAE684-treated samples. The images represent at least ten parasites per sample. Scale bars correspond to 2 µm. **(B)** A representative image of a non-activated mature gametocyte. Scale bar = 5 µm.

1. **Methods**

Parasitology work and volunteer human blood donation (from consenting, healthy adult volunteers) at the University of Pretoria is covered under ethical approval from the Health Sciences Ethics Committee (506/2018) and Natural and Agricultural Sciences Ethics Committee (180000094). Full methods are provided in the supplementary material.

**2.1. Parasite *in vitro* cultivation**

*P. falciparum* drug sensitive NF54 (*Pf*NF54) and drug-resistant Dd2 (*Pf*Dd2, chloroquine, pyrimethamine and mefloquine resistant) strains were cultured *in vitro* in human O^+^/A^+^ erythrocytes (5% hematocrit) in complete medium at 2–5% parasitemia, 37 °C, shaking at 60 rpm under hypoxic conditions (90% N_2_, 5% O_2_ and 5% CO_2_). Immature and mature gametocytes were produced from *P. falciparum* 3D7elo1-pfs16-CBG99 (kind gift from Pietro Alano, ISS, Italy) [^[1]^](https://sciwheel.com/work/citation?ids=11023357&pre=&suf=&sa=0&dbf=0) as described[^[2,3]^](https://sciwheel.com/work/citation?ids=11023296,17940641&pre=&pre=&suf=&suf=&sa=0,0&dbf=0&dbf=0) by simultaneously applying nutrient starvation and decreasing hematocrit to induce >97% ring-stage ABS parasites (0.5% parasitemia, 6% hematocrit). Gametocytogenesis was followed with daily media changes (including glucose) and ABS parasites removed with 50 mM N-acetylglucosamine (day 1–4 for stage II/III; days 3–7 for stage IV/V).

**2.2. *In vitro* asexual and gametocyte activity evaluation**

All inhibitors were dissolved in dimethyl sulfoxide (DMSO). Anti*plasmodium* activity of selected anti-cancer inhibitors was determined on *P. falciparum* NF54 and Dd2 strains, measuring SYBR Green I fluorescence (485 nm excitation, 538 nm emission) as an indicator of proliferation[^[4]^](https://sciwheel.com/work/citation?ids=17919407&pre=&suf=&sa=0&dbf=0). Ring-stage ABS parasites (1% parasitemia, 1% hematocrit) were treated with compounds for 96 h at 37 °C with chloroquine disulphate (0.5 μM) as positive drug control. The activity of the inhibitors was tested against immature (>95% stage II/III) and late-stage (>90% stage IV/V) gametocytes using a luciferase reporter assay on 3D7elo1-pfs16-CBG99 [^[1]^](https://sciwheel.com/work/citation?ids=11023357&pre=&suf=&sa=0&dbf=0). Cultures (2% gametocytemia, 1.5% hematocrit) were exposed to drug pressure for 48 h with methylene blue (5 μM) as a positive control for inhibition. Luciferase activity was determined using a non-lysing D-luciferin substrate (1 mM in 0.1 M citrate buffer, pH 5.5, 100 μL) and bioluminescence was detected with a GloMax® - Multi Detection System with Instinct® software. For all, assay performance was monitored with Z-factors > 0.8 and percentage inhibition was calculated, and dose-response curves were constructed in GraphPad Prism v10.5 to calculate IC_50_ values.

**2.3. *P. berghei* (PbLuc) liver stage screening**

*Plasmodium berghei* (Pb) sporozoites were obtained by dissecting the salivary glands of infected *Anopheles stephensi* mosquitoes. These were provided by The SporoCore, at the University of Georgia, GA, USA ([SporoCore.uga.edu](https://urldefense.com/v3/__http:/sporocore.uga.edu/__;!!LLK065n_VXAQ!g2xX2D5W424B8jlws18hT5fB7c9jdv36_sXyVKtgLIupQCFi-FR8uiAt06vXJDKQLe0inCxpB-6mg9hFwkPd$)). The parasites utilised a GFP-Luc_ama1-eef1_ reporter line[^[5]^](https://sciwheel.com/work/citation?ids=17919413&pre=&suf=&sa=0&dbf=0). These engineered parasites, termed Pb-Luc, were used to infect HepG2-A16-CD81EGFP cells. HepG2-A16-CD81EGFP cells stably transformed to express a GFP-CD81 fusion protein[^[6]^](https://sciwheel.com/work/citation?ids=785427&pre=&suf=&sa=0&dbf=0) were cultured at 37 °C in 5% CO_2_ in DMEM (Invitrogen, Carlsbad, USA) supplemented with 10% FBS (Corning, NY, USA), 1X Pen/Strep/Glu (Thermo Fisher Scientific, USA). Compounds were prepared as 10 mM solutions in DMSO, and 10 nL of each (resulting in a final DMSO concentration of 0.1% per well) was transferred into assay plates using an ECHO 650 (Beckman). Concentrations ranged from 10 µM to 0.5 nM. Atovaquone (0.1 µM) served as a positive control, while 0.1% DMSO was used as a negative control. Liver stage (PbLuc) screening has been previously described[^[7]^](https://sciwheel.com/work/citation?ids=18045749&pre=&suf=&sa=0&dbf=0). Human hepatic cells (3 × 10^3^; HepG2-A16-CD81-EGFP) suspended in 5 µL of DMEM medium (2×10^5^ cells/mL, supplemented with 5% FBS, 5× Pen/Strep/Glu) were seeded in 1536-well plates (Greiner BioOne) using the MultiFlo FX (Agilent) 20 h before infection. *Pb*Luc sporozoites, isolated from the salivary glands of *A. stephensi* mosquitoes, were filtered twice through a 20 µm nylon pore cell strainer. The sporozoites were resuspended in screening media, counted with a hemocytometer, and diluted to a final concentration of 200 sporozoites per µL. Each well received 1,000 sporozoites in 5 µL, dispensed via the GNF Dispenser II (GNF Systems). The plates were then centrifuged for 3 min in an Eppendorf 5810 R (330 RCF) at the lowest acceleration and brake settings. After incubation at 37 °C with 5% CO_2_ for 48 h, the media was removed by centrifuging the inverted plates at 130 RCF for 1 min. Wells then received 2 µL of Bright-Glo™ Luciferase Assay System (Promega). Luminescence was measured immediately with the Pherastar FSX reader (BMG Labtech). Luminescence values were normalized using the positive and negative controls. EC_50_ values were calculated with CDD Vault (Burlingame, CA). All experiments were performed with at least four technical replicates and repeated three times.

**2.4. *P. falciparum* Dual Gamete Formation Assay (*Pf* DGFA)**

The *Pf* DGFA[^[8]^](https://sciwheel.com/work/citation?ids=11567875&pre=&suf=&sa=0&dbf=0) was performed by incubating *Pf*NF54 gametocytes with test molecules in 384-well plates for 48 h. Gametogenesis was triggered with the addition of xanthurenic acid and a decrease in temperature. Male and female gametes were identified through fluorescent microscopy and quantified from recorded data using a custom imaging algorithm and then compared to negative (DMSO) and positive (1 µM cabamiquine) controls. Percentage inhibition was calculated, and dose-response curves were constructed in GraphPad Prism v10.5 to calculate IC_50_ values.

**2.5. Cytotoxicity counter-screening**

HepG2 and CHO cells were maintained at 37 °C, under a humidified atmosphere of 5% CO_2_, in DMEM high glucose media (Gibco) supplemented with 10% heat-inactivated FBS and 1% (v/v) Penicillin-Streptomycin antibiotics. Cells were grown to 90% confluency, after which the cells were washed with PBS and trypsinised with trypsin-EDTA (0.25% Trypsin and 1 mM EDTA, Merck) and centrifuged for 2 min (10 000 x*g*). The supernatant was discarded, and the cell pellet was resuspended in 1 mL DMEM media for cell counting by Trypan Blue. To determine the toxicity, cells were resuspended and seeded at a concentration of 1x10^4^ cells/well in a 96-well plate and incubated for 24 h[^[9,10]^](https://sciwheel.com/work/citation?ids=7014439,17931033&pre=&pre=&suf=&suf=&sa=0,0&dbf=0&dbf=0). Cells were treated in duplicate with a 10-fold serial dilution starting at 100 ug/mL of inhibitor in new media. After 48 h incubation, the viable cells were measured using the MTT tetrazolium reduction assay, where sterile 25 µL of a 5 mg/mL MTT was added to each well and incubated for 4 h before the water-soluble formazan crystals were dissolved with 100 µL DMSO (Merck) per well. The absorbance, a measure of intracellular reduction of MTT, was measured at 540 nm (Multiskan GO). Emetine (50 µM) was included as a positive drug control, and the background was subtracted from all data before being normalised to the untreated[^[9,10]^](https://sciwheel.com/work/citation?ids=7014439,17931033&pre=&pre=&suf=&suf=&sa=0,0&dbf=0&dbf=0).

**2.6. Rate- and stage-specific evaluations and inhibitor reversibility**

To determine the stage specificity during asexual intra-erythrocytic development, *P. falciparum* NF54 parasites were synchronised using 5% D-Sorbitol to obtain a >90% ring-stage population (6–10 h post-invasion, hpi). *In* *vitro*, synchronised parasite cultures were treated at 3xIC_50_ for TAE684, AT83, and ZM-39 and at IC_99_ for hesperadin. The rate of activity and stage-specificity was monitored morphologically using Giemsa-stained thin smears. Asexual intra-erythrocytic stage parasites were treated at ring, early/late trophozoite, and schizont stages, sampling every 12 h over a 48-hour period. Images were captured using a Nikon Eclipse 50i light microscope adapted with a Nikon camera (Nikon DS-Fi1) and NIS-Elements software. To determine how fast the inhibitor affects a specific parasite stage and whether the effect is reversible for each inhibitor, synchronised late trophozoite and schizont stage parasite cultures were treated with TAE684 at 3xIC_50_ and hesperadin at IC_99_ for 12 h, after which the inhibitor was washed off with inhibitor-free medium. Parasite progression was monitored every 12 h over a 48-h period using flow cytometry, as described in[^[11]^](https://sciwheel.com/work/citation?ids=5991606&pre=&suf=&sa=0&dbf=0). The IC_50_ speed assay was conducted as described in[^[12]^](https://sciwheel.com/work/citation?ids=13127339&pre=&suf=&sa=0&dbf=0). Briefly, unsynchronised *P. falciparum* NF54 asexual proliferation was determined using a SYBR Green I proliferation-based assay and expressed as IC_50_ values as described earlier. Four incubation times were employed for each inhibitor: 96 (standard assay time), 72, 48 and 24 h. IC_50_ values were obtained from corrected dose-response curves using Graph-Pad Prism v10.5.

**2.7. Inhibitor susceptibility assays using *P. falciparum* kinase cKD lines**

Compound susceptibility assays using *P. falciparum* Ark1 and Ark2 cKD lines were carried out as previously described[^[13]^](https://sciwheel.com/work/citation?ids=1359778&pre=&suf=&sa=0&dbf=0). Ark1 (PF3D7_0605300) and Ark2 (PF3D7_0309200) cKD lines generated by fusing the coding sequence and non-coding RNA aptamer sequences in the 5’- and 3’-UTR, permitting translation regulation using the TetR-DOZI system[^[14]^](https://sciwheel.com/work/citation?ids=3173621&pre=&suf=&sa=0&dbf=0). Editing was achieved by CRISPR/SpCas9 using the linear pSN054 vector that contains cloning sites for the left homology region (LHR) and the right homology region (RHR), as well as a gene-specific guide RNA under control of the T7 promoter[^[15]^](https://sciwheel.com/work/citation?ids=10322119&pre=&suf=&sa=0&dbf=0). The final constructs were sequence-verified and further confirmed by restriction digests. Transfection into Cas9- and T7 RNA polymerase-expressing NF54 parasites was carried out by pre-loading erythrocytes with the donor vector as previously described[^[16]^](https://sciwheel.com/work/citation?ids=3416697&pre=&suf=&sa=0&dbf=0). Parasite culture was maintained continuously in 500 nM anhydrotetracycline (aTc, Sigma-Aldrich 37919) and drug selection with 2.5 μg/mL of Blasticidin S (RPI Corp B12150-0.1) was initiated four days after transfection. Briefly, synchronous ring-stage Ark1 (PF3D7_0605300) and Ark2 (PF3D7_0309200) cKD parasites, as well as a control parasite line expressing an aptamer-regulatable fluorescent protein, were maintained in the presence of high aTc (500 nM) or no aTc and distributed into 384-well polystyrene microplates (Corning). Stock solutions of compounds were serially diluted and transferred to the parasite-containing plates using the Janus platform (PerkinElmer). DMSO and dihydroartemisinin treatment (500 nM) served as reference controls. Luminescence was measured after 72 hours using the Renilla-Glo Luciferase Assay System (Promega E2750) and the GloMax Discover Multimode Microplate Reader (Promega), and IC_50_ values were obtained from corrected dose-response curves using Graph-Pad Prism v10.5.

**2.8. Antimalarial resistome barcode sequencing (AReBar) cross-resistance assay**

Cross-resistance of Hesperadin, TAE684 and AT9283 was evaluated against a pool of barcoded drug-resistant parasites[^[17]^](https://sciwheel.com/work/citation?ids=13480193&pre=&suf=&sa=0&dbf=0). The pool consisted of 52 lines (**Supplementary File S2**) in both the Dd2 and 3D7 backgrounds, including wild-type, that were barcoded at the *pfpare* locus (PF3D7_0709700)[^[18]^](https://sciwheel.com/work/citation?ids=4395252&pre=&suf=&sa=0&dbf=0), with unique 11-bp barcode sequences. The pool (triplicate cultures of 1 mL) was exposed to 3 xIC_50_ of each test compound, including the positive control compound halofuginone that targets prolyl tRNA synthetase. Growth of the pool was measured every 2–3 days by flow cytometry (Beckman CytoFlex 5), staining with 1xSYBR Green I and 200 nM MitoTracker Deep Red, and parasitemia maintained within a range <5% parasitemia. At day 14, samples were harvested, lysed with 0.05% saponin and parasite pellets collected. Barcodes were amplified by PCR and quantified by sequencing using an Oxford Nanopore minION. The change in barcode proportion (log_2_ fold change, LFC) was measured relative to the no-drug control, with LFC>2.5 indicating cross-resistance.

**2.9. *Pf*PKG, *Pv*PI4Kβ and *Pf*CLK3 Kinase Assays**

Full-length *Pf*PKG (PF3D7_1436600)[^[19]^](https://sciwheel.com/work/citation?ids=4442697&pre=&suf=&sa=0&dbf=0) and *Pv*PI4Kβ (PVX_098050)[^[20]^](https://sciwheel.com/work/citation?ids=123441&pre=&suf=&sa=0&dbf=0) recombinant proteins were expressed in *E. coli* and baculovirus-insect cell expression systems. The kinase domain of *Pf*CLK3 (PF3D7_1114700, Gln317 to Ser694) was expressed in *E. coli* Rosetta™(DE3)pLysS cells (kind gift from Rafael M. Couñago, CQMED, University of Campinas). Briefly, the N-terminal His-tagged kinases were purified using immobilized metal affinity chromatography, followed by anion exchange chromatography in the case of *Pf*PKG, and size exclusion chromatography. Dose-response kinase inhibition assays were carried out using the ADP-Glo kinase assay kit[^[21]^](https://sciwheel.com/work/citation?ids=13869337&pre=&suf=&sa=0&dbf=0), with 1 nM *Pf*PKG, 10 μM ATP, 20 μM GRTGRRNSI-NH_2_, 1% (v/v) DMSO and inhibitor in PKG assay buffer (25 mM HEPES pH 7.4, 0.1 mg/mL BSA, 0.01% (v/v) Triton-X 100, 20 mM MgCl_2_, 2 mM DTT, 10 μM cGMP); or 6 nM *Pv*PI4Kβ, 10 μM ATP, 0.1 mg/ml L-alpha-phosphatidylinositol, 1% (v/v) DMSO and inhibitor in PI4Kβ assay buffer (25 mM HEPES pH 7.4, 100 mM NaCl, 3 mM MgCl2, 1 mM DTT, 0.025 mg/ml BSA, 0.2% (v/v) Triton-X-100); or 15 nM *Pf*CLK3, 10 μM ATP, 20 μM myelin basic protein, 1% (v/v) DMSO and inhibitor in assay buffer (50 mM HEPES pH 7.4, 0.1 mg/mL BSA, 0.01% (v/v) Triton-X 100, 20 mM MgCl2, 2 mM DTT). The data were normalized based on the 100% activity controls (1% DMSO) and the 100% inhibition controls [ML10 for *Pf*PKG, MLN0128 (sapanisertib[^[21]^](https://sciwheel.com/work/citation?ids=13869337&pre=&suf=&sa=0&dbf=0)) for *Pv*PI4Kβ and TCMDC-135051[^[22]^](https://sciwheel.com/work/citation?ids=7646216&pre=&suf=&sa=0&dbf=0) for *Pf*CLK3, at 10 µM]. Mean IC_50_ values were calculated from two independent experiments, each with technical duplicates.

**2.10. Molecular docking**

A homology model for *P. falciparum* Ark1 (*Pf*Ark1) was generated using the crystal structure of human Aurora kinase A (*Hs*AurA) co-crystallized with ATP (PDB 5DNR)[^[23]^](https://sciwheel.com/work/citation?ids=1533478&pre=&suf=&sa=0&dbf=0), with a 33% sequence identity. Model quality parameters included an acceptable Ramachandran analysis with 94% of residues in favored regions, no bad bonds or stereochemical violations, with a root mean square deviation (RMSD) of 0.44 Å. The protein sequences of the *Pf*Ark1, *Hs*AurA, *Hs*AurB and *X. laevis* AurB (*Xen*AurB) were aligned using the NCBI alignment tool[^[24]^](https://sciwheel.com/work/citation?ids=708846&pre=&suf=&sa=0&dbf=0). Ligands were prepared on the Schrödinger 2021-4 Maestro interface using the LigPrep tool at pH 7.4 ± 0.5 using the OPLS4 forcefield, while the crystal was prepared using the Schrödinger Protein Preparation Wizard. Structural optimization included bond order assignments, generation of tautomer and ionization states, addition of hydrogen atoms, side chains and loops, removal of water molecules from the surface of the crystal structure and restrained minimization at pH 7.4 with the default parameters to generate the lowest energy conformation using the OPLS4 forcefield[^[25,26]^](https://sciwheel.com/work/citation?ids=11178572,2592307&pre=&pre=&suf=&suf=&sa=0,0&dbf=0&dbf=0). The receptor grid was generated for the refined *Pf*Ark1 homology model structures by centering a 10 Å inner grid box and a 20 Å outer grid box over specified key residues found in the hinge region (Phe107-The119). Ligands were docked to the receptor using the Glide default settings in extra precision, flexible ligand mode, and poses were scored using the Schrödinger GlideScore function[^[27]^](https://sciwheel.com/work/citation?ids=230404&pre=&suf=&sa=0&dbf=0). Following this, further analysis of the docking poses was conducted using Molecular Mechanics and Generalized Born Surface Area (MM-GBSA) with the default solvation model (VSGB and OPLS4) settings, and residues within 20 Å of the active site were allowed to be flexible[^[28]^](https://sciwheel.com/work/citation?ids=5697396&pre=&suf=&sa=0&dbf=0). Docking poses were visualized and images were generated in Schrödinger 2021-4 Maestro interface.

**2.11. Isobologram analysis**

Fixed-ratio isobologram analysis[^[29]^](https://sciwheel.com/work/citation?ids=1312695&pre=&suf=&sa=0&dbf=0) was performed on parasites were treated with fixed ratios (5:0, 4:1, 3:2, 2:3, 1:4, and 0:5) of TAE684 and hesperadin in combination with chloroquine (CQ), ML10, and each other. Similarly, this was also done for AT83 and ZM-39 in combination with CQ and each other using the SYBR Green I proliferative assay as readouts to determine the fractional inhibitory concentrations (FIC) for the respective combinations. Isobolograms were generated by plotting paired FIC values linearly, utilizing the average of three biological replicates, each performed in technical duplicates. The paired FIC values for each drug combination were analyzed, and the mean FIC values (ΣFIC) were calculated to delineate synergism (<0.8), additivity/indifference (0.8–1.4), or antagonism (>1.4).

**2.12. NP-40 mediated cell-free β-hematin and hemozoin inhibition assays**

The β-hematin inhibition assay, as described in[^[30,31]^](https://sciwheel.com/work/citation?ids=5496816,17309116&pre=&pre=&suf=&suf=&sa=0,0&dbf=0&dbf=0) was used to test the inhibitors for their ability to inhibit β-hematin formation. The test samples were prepared as 20 mM or 10 mM stock solutions in dimethyl sulfoxide (DMSO). Serial dilutions of each inhibitor (100 μL) were performed from column 12 to column 2 of a 96-well plate in triplicate, with column 1 serving as a negative control (0 μM test inhibitor). The samples were tested at a starting in-well concentration of 1000 μM (20 mM stock) or 500 μM (10 mM stock). An “NP-40 substitute” detergent was added (305.5 μM) to each well to mimic the lipophilic environment in which hemozoin formation occurs within the parasite’s digestive vacuole, in helping to mediate the formation of β-hematin. A 25 mM hematin stock was prepared by sonicating heme in DMSO and then suspending 178.8 μL of this in 1 M acetate buffer (20 mL, pH 4.8). Then, 100 μL of the hematin suspension was added to each well to a final well volume of 200 μL. The plate was then incubated for 5 hours at 37 °C. After incubation, 32 μL and 60 μL of 50% pyridine solution and acetone were added, respectively. The pyridine-ferrochrome method by[^[31]^](https://sciwheel.com/work/citation?ids=17309116&pre=&suf=&sa=0&dbf=0) was used for UV-vis analysis, with the heme-pyridine complex absorbance measured at 405 nm. Data were analysed using Microsoft Excel and GraphPad Prism v10.5 software.

Heme fractionation assay:

The assay set-up followed that described in[^[30,32]^](https://sciwheel.com/work/citation?ids=13807560,5496816&pre=&pre=&suf=&suf=&sa=0,0&dbf=0&dbf=0) optimised to a multi-well colourimetric assay for determining heme species in *P. falciparum*. Briefly, *P. falciparum* ring-staged parasites were incubated at 2xIC_50_ value of the test inhibitor for 24 hours. Thereafter, the incubated parasites (trophozoites) were harvested and isolated through various steps of a cellular fractionation process. The cellular fractionations allow for direct quantification of the three major heme species in the trophozoites, namely hemoglobin, free heme, and hemozoin. These heme species can be determined spectroscopically using the aqueous pyridine-ferrochrome method. This method is based on the principle that aqueous pyridine forms a low-spin complex with heme but not hemozoin, and since the absorbance obeys Beer’s law, it allows for the quantification of heme concentration in solution. The various heme fractions were recovered and measured as follows, after the *P. falciparum* trophozoite cells were harvested following exposure to probe inhibitors:

Hemoglobin fraction:

Water (100 µL) was added to lyse the cells, and the suspension was sonicated for 5 min. This was followed by the addition of 50 µL 0.2M HEPES buffer pH 7.5, and then centrifugation at 3600 rpm for 20 min. The resulting supernatant (containing hemoglobin) was then transferred into an adjacent set of wells on the same plate. To which 50 µL of each of 4% SDS, 0.3 M NaCl and 25% pyridine were added, respectively. Thereafter, 200 µL of the resulting solution (400 µL) was transferred to a set of wells on a separate 96-well plate for UV-vis analysis[^[32,33]^](https://sciwheel.com/work/citation?ids=13807560,7472201&pre=&pre=&suf=&suf=&sa=0,0&dbf=0&dbf=0).

Heme fraction:

The pellet from the centrifugation step described above consists of heme and hemozoin. Notably, it is known that pyridine dissolves heme to form a low-spin complex, while the hemozoin remains and can be spun out. Hence, to the pellet, 50 µL of water and 50 µL 4% SDS was added, resuspended and sonicated for 5 min. Then 50 µL each of HEPES 0.2 M pH 7.5, 0.3 M NaCl, and 25% pyridine were added, respectively. The mixture was centrifugated at 3600 rpm and the supernatant (250 µL) was transferred to adjacent wells on a separate 96-well plate. Thereafter, 150 µL of water was added and 200 µL of the mixture transferred to a set of wells on a separate plate for UV-vis analysis[^[32,33]^](https://sciwheel.com/work/citation?ids=7472201,13807560&pre=&pre=&suf=&suf=&sa=0,0&dbf=0&dbf=0).

Hemozoin fraction:

To the remaining pellet, 50 µL each of water and 0.3 M NaOH were added, and the mixture was sonicated for 15 min. After 30 min of incubation at room temperature, 50 µL each of HEPES 0.2 M pH 7.5, 0.3 M HCl, and 25% pyridine were added, respectively. To the resulting mixture (250 µL), 150 µL of water was then added, and 200 µL of this was transferred to a set of wells on a separate 96-well plate for UV-vis analysis[^[32,33]^](https://sciwheel.com/work/citation?ids=13807560,7472201&pre=&pre=&suf=&suf=&sa=0,0&dbf=0&dbf=0).

Cell counting using flow cytometry

Cell counts for the assay were performed using the flow cytometry method described in[^[33]^](https://sciwheel.com/work/citation?ids=7472201&pre=&suf=&sa=0&dbf=0). Cell counting determines the number of trophozoites per sample and, therefore, allows for the quantification of the amount of heme found in each individual cell.

**2.13. Fluorescence and U-ExM microscopy**

*In vitro*, synchronised ring-stage parasite cultures were treated at 3xIC_50_ for TAE684, AT83, and ZM-39 and at IC_99_ for hesperadin and sampled every 12 hours over a 60-hour period. Parasites were then evaluated as follows:

MitoTracker Viability:

For parasite viability, cells were then stained for 30 min with 150 nM MitoTracker Orange CMTMRos (M7510, ThermoFisher) and fixed using 4% paraformaldehyde (PFA), 0.075% glutaraldehyde (GA) for 15 min at 37 °C. Finally, cells were mounted on a glass slide using ProLong™ glass antifade mount medium with NucBlue™ (P36981, ThermoFisher). Images were captured using an EVOS M5000 (ThermoFisher).

Immunofluorescence:

Nuclear content and microtubule morphology were assessed using direct immunofluorescence imaging on fixed parasites (4% PFA, 15 min) on poly-D-lysine-coated coverslips. These were washed three times with PBS, permeabilised with fresh 0.1% Triton X-100, washed another three times with PBS, and blocked with 3% BSA-PBS for 1 h at room temperature (RT). Coverslips were then exposed to 1:500 dilution primary anti-tubulin, mouse monoclonal (Merck, T5192) overnight at 4 °C. Primary antibody was subsequently washed off three times with PBS and incubated in anti-mouse conjugated CF488A secondary antibody produced in chicken (Merck, SAB4600238) for 1 h at RT. Cells were then mounted on a glass slide using ProLong™ glass antifade mount medium with NucBlue™ (P36981, ThermoFisher). Membrane morphology and nuclear material segregation were assessed using fixed parasites (4% PFA, 0.075% GA for 15 min) on poly-D-lysine-coated coverslips. Coverslips were washed three times with PBS and incubated overnight at RT with 5 µM BODIPY-TR-ceramide (D7540, ThermoFisher). Cells were then mounted on a glass slide using ProLong™ glass antifade mount medium with NucBlue™ (P36981, ThermoFisher).

Image acquisition:

Images were acquired using a Zeiss LSM780 Inverted Confocal Laser Scanning Microscope (LSM) (Zeiss, Germany) for super-resolution imaging in the appropriate channels with a ×100 oil-immersion objective and 1.4 numerical aperture. Images were processed using Zeiss ZEN Lite Blue Edition software (Zeiss, Germany) and Fiji software.

Expansion Microscopy:

For ultra expansion microscopy (U-ExM), the procedure was performed as previously described[^[34,35]^](https://sciwheel.com/work/citation?ids=17919676,16224798&pre=&pre=&suf=&suf=&sa=0,0&dbf=0&dbf=0). In short, tightly synchronised *Pf*NF54 cultures were treated with hesperadin (3 µM) and harvested at ±46 hpi. Parasite cultures (0.5% hematorcrit) were then seeded onto and incubated for 30 min at 37 °C on 12 mm round Coverslips, treated with poly-D-lysine for 1 h at 37 °C, in the wells of a 12-well plate. Culture supernatants were removed, and cultures were fixed with 1 mL of 4% v/v PFA in 1× PBS for 15 min at 37 °C. Following fixation, coverslips were washed three times with 37 °C PBS before being treated with 1 mL of 1.4% v/v formaldehyde/2% v/v acrylamide (FA/AA) in PBS. Samples were then incubated at 37 °C overnight. Monomer solution (19% w/w sodium acrylate, 10% v/v acrylamide, 0.1% v/v N,N’-methylenebisacrylamide in PBS) was made the night before gelation and stored at −20 °C overnight. Prior to gelation, FA/AA solution was removed from coverslips, and they were washed once in PBS. For gelation, 5 µL of 10% v/v tetraethylenediamine (TEMED) and 5 µL of 10% w/v ammonium persulfate (APS) were added to 90 µL of monomer solution and briefly vortexed. Subsequently, 35 µL was pipetted onto parafilm, and coverslips were placed (cell side down) on top. Gels were incubated at 37 °C for 30 min before being transferred to wells of a 6-well plate containing denaturation buffer (200 mM SDS, 200 mM NaCl, 50 mM Tris, pH 9). Gels were incubated in denaturation buffer with shaking for 15 min, before the separated gels were transferred to 1.5 mL tubes containing denaturation buffer. 1.5 mL tubes were incubated at 95 °C for 90 min. Following denaturation, the gels were transferred to 10 cm Petri dishes containing 25 mL of MilliQ water for the first round of expansion and placed on a shaker for 30 min three times, with the water changed between each time. Gels were subsequently shrunk with two 15 min washes in 25 mL of 1× PBS, before being transferred to 6-well plates for 30 min of blocking in 3% BSA-PBS at room temperature. After blocking, the gels were incubated overnight with primary antibodies diluted in 3% BSA-PBS. After primary antibody incubation, gels were washed three times in 0.5% v/v PBS-Tween 20 for 10 min before incubation with secondary antibodies diluted in 1× PBS for 2.5 h. Following secondary antibody incubation, gels were again washed three times in PBS-Tween 20, before being transferred back to 10 cm Petri dishes for re-expansion with three 30 min MilliQ water incubations. Gels were either imaged immediately following re-expansion or stored in 0.2% w/v propyl gallate in MilliQ water until imaging.

Image acquisition:

A small slice of gel (~10 × 10 mm) was cut and mounted on an imaging dish (35 mm Cellvis coverslip-bottomed dishes, Fisher Scientific) coated with poly-D-lysine. The side of the gel containing the sample is placed face down on the coverslip, and a few drops of MiliQ H_2_O are added after mounting to prevent gel shrinkage due to dehydration during imaging. All images presented in this study were captured using a Zeiss LSM980 microscope with an AxioObserver and an Airyscan 2 detector. Imaging was conducted on both microscopes using a ×63 Plan-Apochromat objective lens with a numerical aperture of 1.4. All images were acquired as Z-stacks that had an XY pixel size of 0.035 µm and a Z-slice size of 0.13 µm. Images were processed using Zeiss ZEN Lite Blue Edition software (Zeiss, Germany) and Fiji software.

Microtubule branch length image processing:

Rolling ball background subtraction and Gaussian blur were first applied to the image channel to reduce noise. The microtubule branches were then semi-automatically traced using the FIJI Simple Neurite Tracer (SNT) software V4.2.1.[^[36]^](https://sciwheel.com/work/citation?ids=10820460&pre=&suf=&sa=0&dbf=0).

**2.14. Statistical analysis**

A two-tailed t-test (95% CI) was used for the statistical assessment of measurement differences that might present significance relative to the control. The significant differences are displayed as asterisks on graphs: **p*<0.05; ***p*<0.01; ****p*<0.001, *****p*<0.0001. The data presented was from one experiment performed in quadruplicate and subsequently analyzed using Microsoft Excel and GraphPad Prism v10.5 software.

**2.15. In vitro ADME**

In vitro ADME analysis for logD, kinetic solubility (PBS, pH 7.4), human liver microsome clearance and t_1/2_, rat hepatocyte clearance and t_1/2_, and protein binding in Albumax media (simulating the malaria parasite culture media) was all performed as per standard assays by TCG LifeSciences.

**References**

[[1]    L. Cevenini, G. Camarda, E. Michelini, G. Siciliano, M. M. Calabretta, R. Bona, T. R. S. Kumar, A. Cara, B. R. Branchini, D. A. Fidock, A. Roda, P. Alano, *Anal. Chem.* **2014**, *86*, 8814–8821.](https://sciwheel.com/work/bibliography/11023357)

[[2]    J. Reader, M. Botha, A. Theron, S. B. Lauterbach, C. Rossouw, D. Engelbrecht, M. Wepener, A. Smit, D. Leroy, D. Mancama, T. L. Coetzer, L.-M. Birkholtz, *Malar. J.* **2015**, *14*, 213.](https://sciwheel.com/work/bibliography/11023296)

[[3]    J. Reader, M. E. van der Watt, L.-M. Birkholtz, *Front. Cell. Infect. Microbiol.* **2022**, *12*, 926460.](https://sciwheel.com/work/bibliography/17940641)

[[4]    J. Reader, M. E. van der Watt, D. Taylor, C. Le Manach, N. Mittal, S. Ottilie, A. Theron, P. Moyo, E. Erlank, L. Nardini, N. Venter, S. Lauterbach, B. Bezuidenhout, A. Horatscheck, A. van Heerden, N. J. Spillman, A. N. Cowell, J. Connacher, D. Opperman, L. M. Orchard, M. Llinás, E. S. Istvan, D. E. Goldberg, G. A. Boyle, D. Calvo, D. Mancama, T. L. Coetzer, E. A. Winzeler, J. Duffy, L. L. Koekemoer, G. Basarab, K. Chibale, L.-M. Birkholtz, *Nat. Commun.* **2021**, *12*, 269.](https://sciwheel.com/work/bibliography/17919407)

[[5]    A. K. Pathak, J. C. Shiau, B. Franke-Fayard, L. M. Shollenberger, D. A. Harn, D. E. Kyle, C. C. Murdock, *Malar. J.* **2022**, *21*, 264.](https://sciwheel.com/work/bibliography/17919413)

[[6]    S. Yalaoui, S. Zougbédé, S. Charrin, O. Silvie, C. Arduise, K. Farhati, C. Boucheix, D. Mazier, E. Rubinstein, P. Froissard, *PLoS Pathog.* **2008**, *4*, e1000010.](https://sciwheel.com/work/bibliography/785427)

[[7]    J. Swann, V. Corey, C. A. Scherer, N. Kato, E. Comer, M. Maetani, Y. Antonova-Koch, C. Reimer, K. Gagaring, M. Ibanez, D. Plouffe, A.-M. Zeeman, C. H. M. Kocken, C. W. McNamara, S. L. Schreiber, B. Campo, E. A. Winzeler, S. Meister, *ACS Infect. Dis.* **2016**, *2*, 281–293.](https://sciwheel.com/work/bibliography/18045749)

[[8]    M. J. Delves, U. Straschil, A. Ruecker, C. Miguel-Blanco, S. Marques, A. C. Dufour, J. Baum, R. E. Sinden, *Nat. Protoc.* **2016**, *11*, 1668–1680.](https://sciwheel.com/work/bibliography/11567875)

[[9]    B. K. Verlinden, J. Niemand, J. Snyman, S. K. Sharma, R. J. Beattie, P. M. Woster, L.-M. Birkholtz, *J. Med. Chem.* **2011**, *54*, 6624–6633.](https://sciwheel.com/work/bibliography/7014439)

[[10]   M. Leshabane, G. A. Dziwornu, D. Coertzen, J. Reader, P. Moyo, M. van der Watt, K. Chisanga, C. Nsanzubuhoro, R. Ferger, E. Erlank, N. Venter, L. Koekemoer, K. Chibale, L.-M. Birkholtz, *ACS Infect. Dis.* **2021**, *7*, 1945–1955.](https://sciwheel.com/work/bibliography/17931033)

[[11]   R. van Biljon, J. Niemand, R. van Wyk, K. Clark, B. Verlinden, C. Abrie, H. von Grüning, W. Smidt, A. Smit, J. Reader, H. Painter, M. Llinás, C. Doerig, L.-M. Birkholtz, *Sci. Rep.* **2018**, *8*, 16581.](https://sciwheel.com/work/bibliography/5991606)

[[12]   C. Le Manach, C. Scheurer, S. Sax, S. Schleiferböck, D. G. Cabrera, Y. Younis, T. Paquet, L. Street, P. Smith, X. C. Ding, D. Waterson, M. J. Witty, D. Leroy, K. Chibale, S. Wittlin, *Malar. J.* **2013**, *12*, 424.](https://sciwheel.com/work/bibliography/13127339)

[[13]   S. J. Goldfless, J. C. Wagner, J. C. Niles, *Nat. Commun.* **2014**, *5*, 5329.](https://sciwheel.com/work/bibliography/1359778)

[[14]   S. M. Ganesan, A. Falla, S. J. Goldfless, A. S. Nasamu, J. C. Niles, *Nat. Commun.* **2016**, *7*, 10727.](https://sciwheel.com/work/bibliography/3173621)

[[15]   A. S. Nasamu, A. Falla, C. F. A. Pasaje, B. A. Wall, J. C. Wagner, S. M. Ganesan, S. J. Goldfless, J. C. Niles, *Sci. Rep.* **2021**, *11*, 342.](https://sciwheel.com/work/bibliography/10322119)

[[16]   K. Deitsch, C. Driskill, T. Wellems, *Nucleic Acids Res.* **2001**, *29*, 850–853.](https://sciwheel.com/work/bibliography/3416697)

[[17]   M. Carrasquilla, N. F. Drammeh, M. Rawat, T. Sanderson, Z. Zenonos, J. C. Rayner, M. C. S. Lee, *MBio* **2022**, *13*, e0093722.](https://sciwheel.com/work/bibliography/13480193)

[[18]   E. S. Istvan, J. P. Mallari, V. C. Corey, N. V. Dharia, G. R. Marshall, E. A. Winzeler, D. E. Goldberg, *Nat. Commun.* **2017**, *8*, 14240.](https://sciwheel.com/work/bibliography/4395252)

[[19]   D. A. Baker, L. B. Stewart, J. M. Large, P. W. Bowyer, K. H. Ansell, M. B. Jiménez-Díaz, M. El Bakkouri, K. Birchall, K. J. Dechering, N. S. Bouloc, P. J. Coombs, D. Whalley, D. J. Harding, E. Smiljanic-Hurley, M. C. Wheldon, E. M. Walker, J. T. Dessens, M. J. Lafuente, L. M. Sanz, F.-J. Gamo, S. B. Ferrer, R. Hui, T. Bousema, I. Angulo-Barturén, A. T. Merritt, S. L. Croft, W. E. Gutteridge, C. A. Kettleborough, S. A. Osborne, *Nat. Commun.* **2017**, *8*, 430.](https://sciwheel.com/work/bibliography/4442697)

[[20]   C. W. McNamara, M. C. Lee, C. S. Lim, S. H. Lim, J. Roland, O. Simon, B. K. Yeung, A. K. Chatterjee, S. L. McCormack, M. J. Manary, A.-M. Zeeman, K. J. Dechering, T. S. Kumar, P. P. Henrich, K. Gagaring, M. Ibanez, N. Kato, K. L. Kuhen, C. Fischli, A. Nagle, M. Rottmann, D. M. Plouffe, B. Bursulaya, S. Meister, L. Rameh, J. Trappe, D. Haasen, M. Timmerman, R. W. Sauerwein, R. Suwanarusk, B. Russell, L. Renia, F. Nosten, D. C. Tully, C. H. Kocken, R. J. Glynne, C. Bodenreider, D. A. Fidock, T. T. Diagana, E. A. Winzeler, *Nature* **2013**, *504*, 248–253.](https://sciwheel.com/work/bibliography/123441)

[[21]   L. B. Arendse, J. M. Murithi, T. Qahash, C. F. A. Pasaje, L. C. Godoy, S. Dey, L. Gibhard, S. Ghidelli-Disse, G. Drewes, M. Bantscheff, M. J. Lafuente-Monasterio, S. Fienberg, L. Wambua, S. Gachuhi, D. Coertzen, M. van der Watt, J. Reader, A. S. Aswat, E. Erlank, N. Venter, N. Mittal, M. R. Luth, S. Ottilie, E. A. Winzeler, L. L. Koekemoer, L.-M. Birkholtz, J. C. Niles, M. Llinás, D. A. Fidock, K. Chibale, *Sci. Transl. Med.* **2022**, *14*, eabo7219.](https://sciwheel.com/work/bibliography/13869337)

[[22]   M. M. Alam, A. Sanchez-Azqueta, O. Janha, E. L. Flannery, A. Mahindra, K. Mapesa, A. B. Char, D. Sriranganadane, N. M. B. Brancucci, Y. Antonova-Koch, K. Crouch, N. V. Simwela, S. B. Millar, J. Akinwale, D. Mitcheson, L. Solyakov, K. Dudek, C. Jones, C. Zapatero, C. Doerig, D. C. Nwakanma, M. J. Vázquez, G. Colmenarejo, M. J. Lafuente-Monasterio, M. L. Leon, P. H. C. Godoi, J. M. Elkins, A. P. Waters, A. G. Jamieson, E. F. Álvaro, L. C. Ranford-Cartwright, M. Marti, E. A. Winzeler, F. J. Gamo, A. B. Tobin, *Science* **2019**, *365*, DOI 10.1126/science.aau1682.](https://sciwheel.com/work/bibliography/7646216)

[[23]   M. Janeček, M. Rossmann, P. Sharma, A. Emery, D. J. Huggins, S. R. Stockwell, J. E. Stokes, Y. S. Tan, E. G. Almeida, B. Hardwick, A. J. Narvaez, M. Hyvönen, D. R. Spring, G. J. McKenzie, A. R. Venkitaraman, *Sci. Rep.* **2016**, *6*, 28528.](https://sciwheel.com/work/bibliography/1533478)

[[24]   S. F. Altschul, J. C. Wootton, E. M. Gertz, R. Agarwala, A. Morgulis, A. A. Schäffer, Y.-K. Yu, *FEBS J.* **2005**, *272*, 5101–5109.](https://sciwheel.com/work/bibliography/708846)

[[25]   C. Lu, C. Wu, D. Ghoreishi, W. Chen, L. Wang, W. Damm, G. A. Ross, M. K. Dahlgren, E. Russell, C. D. Von Bargen, R. Abel, R. A. Friesner, E. D. Harder, *J. Chem. Theory Comput.* **2021**, *17*, 4291–4300.](https://sciwheel.com/work/bibliography/11178572)

[[26]   G. M. Sastry, M. Adzhigirey, T. Day, R. Annabhimoju, W. Sherman, *J. Comput. Aided Mol. Des.* **2013**, *27*, 221–234.](https://sciwheel.com/work/bibliography/2592307)

[[27]   T. A. Halgren, R. B. Murphy, R. A. Friesner, H. S. Beard, L. L. Frye, W. T. Pollard, J. L. Banks, *J. Med. Chem.* **2004**, *47*, 1750–1759.](https://sciwheel.com/work/bibliography/230404)

[[28]   J. Li, R. Abel, K. Zhu, Y. Cao, S. Zhao, R. A. Friesner, *Proteins* **2011**, *79*, 2794–2812.](https://sciwheel.com/work/bibliography/5697396)

[[29]   Q. L. Fivelman, I. S. Adagu, D. C. Warhurst, *Antimicrob. Agents Chemother.* **2004**, *48*, 4097–4102.](https://sciwheel.com/work/bibliography/1312695)

[[30]   J. M. Combrinck, T. E. Mabotha, K. K. Ncokazi, M. A. Ambele, D. Taylor, P. J. Smith, H. C. Hoppe, T. J. Egan, *ACS Chem. Biol.* **2013**, *8*, 133–137.](https://sciwheel.com/work/bibliography/5496816)

[[31]   K. K. Ncokazi, T. J. Egan, *Anal. Biochem.* **2005**, *338*, 306–319.](https://sciwheel.com/work/bibliography/17309116)

[[32]   R. D. Sandlin, M. D. Carter, P. J. Lee, J. M. Auschwitz, S. E. Leed, J. D. Johnson, D. W. Wright, *Antimicrob. Agents Chemother.* **2011**, *55*, 3363–3369.](https://sciwheel.com/work/bibliography/13807560)

[[33]   J. M. Combrinck, K. Y. Fong, L. Gibhard, P. J. Smith, D. W. Wright, T. J. Egan, *Malar. J.* **2015**, *14*, 253.](https://sciwheel.com/work/bibliography/7472201)

[[34]   B. Liffner, S. Absalon, *Microorganisms* **2021**, *9*, DOI 10.3390/microorganisms9112306.](https://sciwheel.com/work/bibliography/17919676)

[[35]   B. Liffner, A. K. Cepeda Diaz, J. Blauwkamp, D. Anaguano, S. Frolich, V. Muralidharan, D. W. Wilson, J. D. Dvorin, S. Absalon, *eLife* **2023**, *12*, DOI 10.7554/eLife.88088.](https://sciwheel.com/work/bibliography/16224798)

[[36]   C. Arshadi, U. Günther, M. Eddison, K. I. S. Harrington, T. A. Ferreira, *Nat. Methods* **2021**, *18*, 374–377.](https://sciwheel.com/work/bibliography/10820460)
